# Neuraminidase 1 Exacerbated Glycolytic Dysregulation and Cardiotoxicity by Destabilizing SIRT1 through Interactions with NRF2 and HIF1α

**DOI:** 10.1002/advs.202414504

**Published:** 2025-05-24

**Authors:** Ting Gao, Yufeng Tang, Tao Zeng, Jie Wang, Xiaohui Zhang, Qingbo Liu, Xun Guan, Xinyu Tang, Guangping Lu, Jiahao Li, Mingrui Liu, Dongmei Zhang, Sixuan Lv, Junlian Gu

**Affiliations:** ^1^ School of Nursing and Rehabilitation Cheeloo College of Medicine Shandong University Jinan Shandong 250012 China; ^2^ Department of Orthopedic Surgery The First Affiliated Hospital of Shandong First Medical University Jinan Shandong 250014 China; ^3^ School of Public Health, Cheeloo College of Medicine Shandong University Jinan Shandong 250012 China

**Keywords:** cardiotoxicity, doxorubicin, glycolysis, metabolic homeostasis, neuraminidase 1

## Abstract

Despite significant therapeutic advances, cumulative DOX‐induced cardiotoxicity (DIC) events remain unacceptably high. Recent evidence has underscored the critical role of impaired glycolytic metabolism in cardiovascular damage. Neuraminidase 1 (NEU1), a member of the neuraminidase family, catalyzes the hydrolysis of terminal sialic acids from glycoconjugates. Here, it is aimed to characterize the role of NEU1 on defective glycolysis during DIC. Mouse models with cardiac‐specific genetic modifications of *Neu1*, *Nrf2*, and *Sirt1* underwent functional analyses, and RNA sequencing to clarify NEU1's role in glycolytic metabolism during DIC. It is discovered that NEU1 is highly expressed after DOX exposure and positively correlated with defective glycolysis phenotypes. Cardiomyocyte‐specific deficiency of *Neu1* ameliorated impaired glycolytic metabolism and DIC, whereas overexpression of *Neu1* in cardiomyocytes exacerbated these pathological phenotypes. Mechanistically, the upregulation of *Neu1* is attributed to HIF1α’s transcriptional repression, which necessitated the collaboration of NRF2. Additionally, the C‐terminal region of NEU1 physically interacted with SIRT1, facilitating its lysosomal‐mediated degradation and contributing to the aberrant glycolytic phenotype. The pharmacological or genetic manipulation of NRF2 and HIF1α remarkably abolished DOX‐induced NEU1 upregulation, compromised glucose metabolism, and DIC progression. Collectively, NEU1 as a key regulator of cardiac glycolysis is established, offering new therapeutic avenues for DIC through maintaining metabolic flexibility.

## Graphical abstract

NEU1, a key regulator of glycolysis, is markedly upregulated following DOX treatment. This upregulation is attributed to HIF1α’s transcriptional repression, requiring intricate interactions with NRF2. Increased NEU1 facilitates SIRT1 lysosomal degradation, contributing to aberrant glycolytic phenotype and cardiac damage. Targeting NEU1 presents a promising clinical strategy to mitigate DOX‐induced cardiotoxicity by preserving metabolic flexibility.

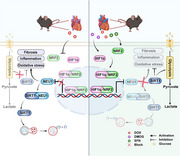

## 1 Introduction

Doxorubicin (DOX) has been widely utilized in various antineoplastic therapies since 1960s, but a robust positive correlation has been increasingly recognized between cumulative DOX dosing and cardiotoxicity.^[^ ^1^, ^2^ ^]^ Currently, dexrazoxane is the only FDA‐approved treatment for DOX‐induced cardiotoxicity (DIC), acting as an iron chelator to reduce cellular damage and lipid peroxidation. However, its use is associated with a poor prognosis and an increased risk of secondary malignancies.^[^ ^3^ ^]^ Other therapies, like adjusted dosing regimens, antioxidants, PEGylated liposomal DOX, and standard heart failure medications (e.g., renin‐angiotensin system inhibitors, β‐blockers), fail to specifically address the molecular pathways involved in DIC. Therefore, it is imperative to explore efficient, specific, and targeted pharmacotherapies for patients to prevent DIC.

Mechanisms postulated for DIC are multifactorial, involving DNA damage, mitochondrial dysfunction, chronic inflammation, oxidative stress, and multiple forms of cell death.^[^ ^4^, ^5^ ^]^ Importantly, recent advances have uncovered a critical role for glycolysis—a metabolic pathway that converts glucose into pyruvate while generating limited amounts of ATP—in the etiology of DOX cardiomyopathy. For example, DOX exposure significantly impaired multiple glycolytic enzyme transcripts, reduced glycolysis rate, diminished cardiac glucose uptake, and altered plasma glucose and lactate levels.^[^ ^3^, ^6^, ^7^, ^8^ ^]^ Furthermore, the suppression of glycolysis by 2‐deoxyglucose (2‐DG) further aggravated DOX‐induced apoptosis, whereas the activation of the key glycolytic enzyme PFKM markedly alleviated DOX‐induced myocardial injury.^[^ ^7^ ^]^ These findings underscore that targeting the glycolysis mechanisms to preserve metabolic elasticity has emerged as a promising therapeutic method to hinder DIC progression. However, the precise molecular mechanism underlying defective glycolysis in DIC remains unknown.

Identification of a novel molecular regulator associated with defective glycolysis is a crucial step strategy in drug discovery to attenuate DIC. Neuraminidases (NEUs), also known as sialidases, are members of the glycosidase family responsible for the removal of terminal α‐glucoside‐linked sialic acid residues from the carbohydrate groups of glycoproteins and glycolipids.^[^ ^9^, ^10^ ^]^ Although NEUs have been demonstrated to exhibit an essential role in multiple human diseases, including neurodegenerative disorders,^[^ ^10^ ^]^ autoimmune,^[^ ^11^ ^]^ cancers,^[^ ^12^ ^]^ lung diseases^[^ ^13^ ^]^ and cardiovascular diseases,^[^ ^14^, ^15^, ^16^ ^]^ the exact biological effects of mammalian NEUs remained less characterized. Based on their subcellular localization and enzymatic characteristics, four NEU isoforms have been identified (NEU1, NEU2, NEU3, and NEU4).^[^ ^14^ ^]^ Among these isoforms, NEU1 is the most abundantly expressed in mammals. Except for its typical catabolic function in lysosomes, NEU1 can translocate to the cell surface, where it modulates the structure and function of cellular receptors.^[^ ^14^, ^17^ ^]^ Previous studies have established an intimate link between NEU1 and disease phenotype in the heart. For instance, Chen et al. demonstrated that NEU1 was remarkably upregulated in patients with hypertrophic cardiomyopathy.^[^ ^14^ ^]^ Simultaneously, after DOX treatment, a phenotype of elevated NEU1 in plasm and cardiac tissue was detected, which exacerbates the DOX‐induced DRP1‐dependent mitochondrial fission and mitophagy.^[^ ^18^ ^]^ Similarly, NEU1 translocated into the nucleus and interacted with transcriptional factor GATA4, leading to cardiomyocyte hypertrophy in a transverse aortic constriction mouse model.^[^ ^14^ ^]^ By contrast, down‐regulation of NEU1 attenuated infiltration of macrophages, excessive mitochondrial fission and mitophagy, ultimately improving atherosclerosis, DIC, and ischemia/reperfusion injury.^[^ ^15^, ^16^, ^18^ ^]^ Moreover, extensive preclinical investigations have illuminated the role of NEU1 in the pathogenesis of various metabolic disorders, including diabetes mellitus, obesity, and non‐alcoholic fatty liver disease, all of which are associated with alterations in glycolytic pathways.^[^ ^19^, ^20^, ^21^, ^22^ ^]^ Therefore, there is a critical need for comprehensive studies to elucidate the mechanisms by which NEU1 may mediate glycolytic dysregulation associated with DIC.

In the present study, we aimed to elucidate the role and potential mechanisms through which NEU1 contributes to DOX‐induced glycolytic disorders. Our findings revealed that NEU1, a critical regulator of impaired glycolysis, was significantly upregulated in DIC. Cardiac‐specific overexpression of *Neu1* disrupted glycolytic metabolism and exacerbated DIC, whereas downregulation of *Neu1* expression in cardiomyocytes effectively mitigated these detrimental phenotypes. Further investigation indicated that DOX‐induced upregulation of NEU1 was due to the transcriptional repression by hypoxia‐inducible factor 1‐alpha (HIF1α), which necessitated the intricate cooperation of NFE2 like bZIP transcription factor 2 (NRF2). We also identified that NEU1 interacted with sirtuin 1 (SIRT1), which was especially subjected to lysosomal but not proteasomal degradation, thereby contributing to abnormal glycolytic phenotype and DIC. These findings not only underscore the critical regulatory role of NEU1 in cardiac glycolytic metabolism but also offer valuable insights into the metabolic flexibility associated with DIC, suggesting NEU1 as a promising target for future therapeutic interventions.

## 2 Results

### 2.1 NEU1 Played a Pivotal Role in Glucose Metabolic Disorders and Cardiac Disease

**Figure 1:**
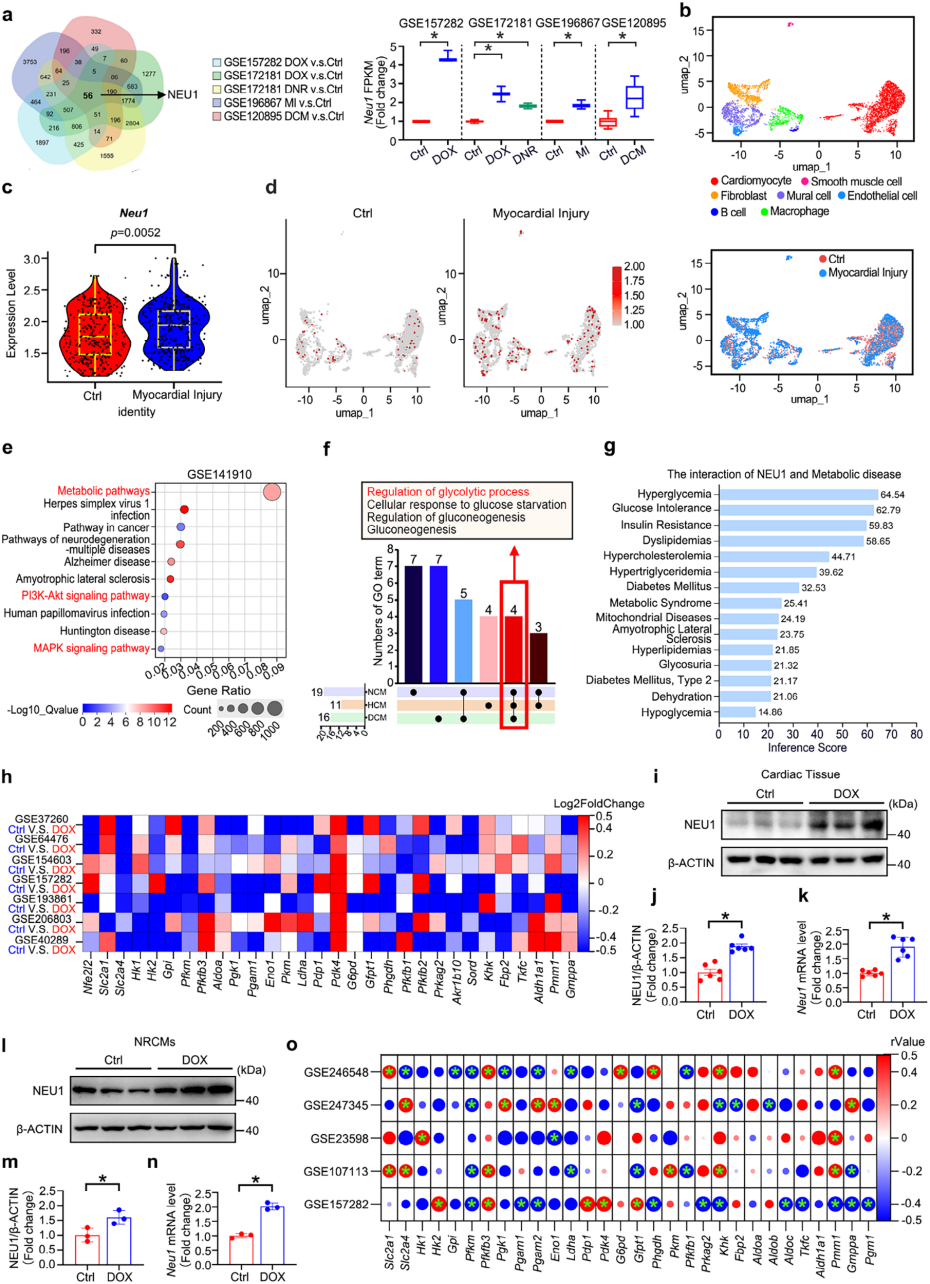
NEU1 is a Critical Regulator of Glucose Metabolism. a) The transcriptional expression of *Neu1* was examined in RNA‐seq data (GSE157282, GSE172181, GSE196867 and GSE120895). (b–d) Analysis of the scRNA‐seq dataset GSE247061: b) UMAP visualization of 5079 cells, colored by cell type (top) and treatment group (bottom). c) Violin plot displaying the expression of the *Neu1* gene across individual cells in different groups. d) UMAP visualization highlighting cells expressing *Neu1*. e) Enrichment analysis of KEGG terms was conducted for *Neu1* co‐expression genes in GSE141910. f) UpSet diagram showing the common GO terms for *Neu1* co‐expression genes among NCM, HCM, and DCM group in GSE141910. g) Interactions between the *Neu1* and metabolic disease based on the CTD. h) Publicly available transcriptomic data from heart tissue or cells treated with DOX were selected to create heat maps showing the expression of genes associated with glycolysis (GSE37260, GSE64476, GSE154603, GSE157282, GSE193861, GSE206803, and GSE40289). The color on the heat map indicates the Log2FoldChange in gene expression compared to the control group. The red color indicates upregulation, while the blue color indicates downregulation. i–k) The protein and mRNA levels of NEU1 in cardiac tissues from mice were detected by WB and RT‐qPCR (*n* = 6). l–n) The protein and mRNA levels of NEU1 in NRCMs were detected by WB and RT‐qPCR (*n* = 3). o) The correlation of *Neu1* and genes related to glucose metabolism. Red: positive correlation. Blue: negative correlation. β‐ACTIN served as an internal control and data are expressed as mean±SD. Additionally, data in (a and c) are presented as box‐and‐whisker plots displaying median and interquartile ranges, *p < 0.05.

To delve deeper into the characteristics of NEU1 in cardiovascular diseases, we analyzed the Comparative Toxicogenomics Database (CTD, http://ctdbase.org/) and RNA‐sequencing (RNA‐seq) data from the public Gene Expression Omnibus (GEO) databases, including GSE157282, GSE172181, GSE196867 and GSE120895. Notably, the expression of *Neu1* transcript was remarkably elevated in damaged cardiomyocytes and tightly associated with a variety of cardiovascular disorders, indicating its crucial role in cardiac pathogenesis (**Figure** **1**a; Figure S1a, Supporting Information). Furthermore, we performed the analysis of single‐cell RNA sequencing (scRNA‐seq) on mouse hearts using the public dataset GSE247061, a total of 5079 cells were obtained and then identified into seven distinct cell clusters (Figure 1b). Consistent with our previous findings, we observed that the expression level of NEU1 in cardiac tissue and the proportion of cells expressing NEU1 increased remarkably after myocardial injury (Figure 1c,d). Given the intricate association between NEU1 and cardiac pathogenesis remaining ambiguous, the expression of *Neu1* and its tight co‐expression genes in non‐failing healthy donors (NCM) tissue, hypertrophic cardiomyopathy (HCM), and dilated cardiomyopathy (DCM) were evaluated by Pearson's correlation analysis. As shown in Figure S1b (Supporting Information), *Neu1*‐associated genes with a p‐value less than 0.05 and an absolute value of correlational coefficient (|r|) greater than 0.5 were subjected to the Kyoto Encyclopedia of Genes and Genomes (KEGG) enrichment analysis. As a result, a significant enrichment of metabolic pathways‐related terms was specifically identified (Figure 1e; Figure S1c, Supporting Information). Subsequently, Gene Ontology (GO) enrichment analysis further emphasized the enrichment of the “Regulation of glycolytic process” in NCM, HCM, and DCM from the GSE141910 dataset (Figure 1f). Consistently, NEU1 is predominantly associated with glucose metabolism‐related diseases after enrichment analysis using the CTD database (Figure 1g). Simultaneously, the GeneMANIA network revealed a high degree of interconnectedness between *Neu1* and multiple key glycolysis‐associated genes (Figure S1d, Supporting Information).

Comprehensive analyses across multiple databases have elucidated that DOX‐based chemotherapies disrupt glycolysis, thereby contributing to metabolic imbalances and disease progression (Figure 1h). In alignment with these findings, our current study revealed a significant increase in the expression levels of NEU1 and a reduction in glycolysis‐related gene expression in primary neonatal rat cardiomyocytes (NRCMs) and mouse heart tissue following DOX administration (Figure 1i–n; Figure S2, Supporting Information). Importantly, our analysis of public GEO databases—specifically GSE246548, GSE247345, GSE23598, GSE107113 and GSE157282—revealed a significant negative correlation between multiple glycolysis‐related genes and *Neu1* in cases of chemotherapeutic‐induced cardiomyopathy (Figure 1o). Collectively, these findings indicate that NEU1 may play a regulatory role in chemotherapy‐induced cardiotoxicity, potentially through the modulation of glycolytic pathways.

### 2.2 NEU1 Impaired Cardiac Glucose Metabolic Phenotype Following DOX Exposure

**Figure 2:**
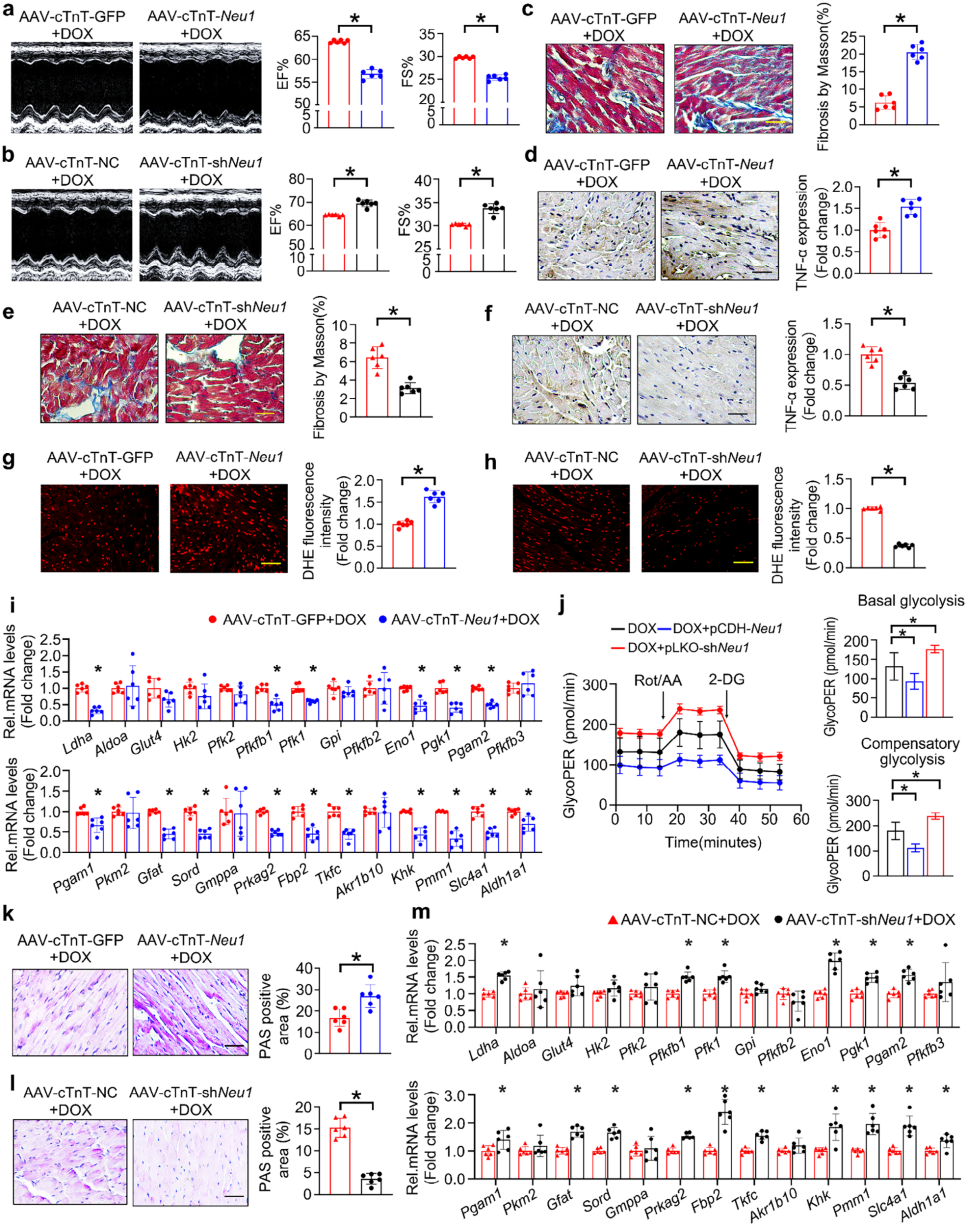
NEU1 Mediates DOX‐induced Glucose Metabolism Disorder and Cardiac Injury. a,b) Mice administrated with AAV9‐cTnT‐GFP, AAV9‐cTnT‐*Neu1*, AAV9‐cTnT‐NC, or AAV9‐cTnT‐sh*Neu1* via tail‐vein injection were subjected to DOX treatment. The cardiac function was assessed by echocardiography (*n* = 6). c,e) The fiber deposition was evaluated by Masson trichrome staining (*n* = 6). d,f) Representative image and quantification of TNF‐α IHC staining (*n* = 6). g,h) The ROS level was detected by DHE staining (*n* = 6). i,m) RT‐qPCR was used to examine the relative mRNA expression levels in the cardiac tissues of mice given AAV9‐cTnT‐GFP, AAV9‐cTnT‐*Neu1*, AAV9‐cTnT‐NC or AAV9‐cTnT‐sh*Neu1* (*n* = 6). j) After being infected with pCDH‐*Neu1* or pLKO‐sh*Neu1*, NRCMs were subjected to DOX (1 µM) treatment for 24 h. The ECAR and OCR were measured sequentially under basal conditions and after treatments of ROT/AA (0.5 µM) and 2‐DG (50 mM). The data were then converted as Glycolytic proton efflux rate (glycoPER) (*n* = 5). k,l) The glycogen deposition was assessed by PAS staining (*n* = 6). Data are expressed as mean±SD, Scale bar = 20 µm in (c–h) and (k–l), *p < 0.05.

To further investigate the exact role of NEU1 in dysregulated glucose homeostasis and DOX‐triggered cardiotoxicity, mice were administered with recombinant adeno‐associated virus serotype 9 (AAV9) vectors carrying *Neu1* or short hairpin RNAs targeting *Neu1* under the cTnT promoter to achieve cardiomyocyte‐specific overexpression or knockdown of *Neu1* via tail‐vein injection, before DOX treatment (Figures S3a and S4a, Supporting Information). The echocardiographic evaluation of left ventricular function and architecture demonstrated that cardiac‐specific overexpression of *Neu1* significantly exacerbated cardiac dysfunction compared with the DOX group, as manifested by a decrement in ejection fraction (EF) and fractional shortening (FS) (**Figure** **2**a). However, these deleterious cardiac functional alterations were conspicuously mitigated by the cardiac‐specific knockdown of the *Neu1* gene (Figure 2b).

Multiple pathomechanisms, including oxidative stress, inflammation, and fibrosis, have been inextricably linked to DIC and ultimately contribute to cardiac dysfunction.^[^ ^23^, ^24^, ^25^ ^]^ Sirius red, Masson staining, and IHC staining for TNF‐α, as well as the mRNA levels of *Tgfb*, *Ctgf*, *Tnfa*, and *Il1b* have revealed more apparent DOX‐induced structural damage and inflammation response following the cardiac‐specific overexpression of *Neu1* (Figure 2c,d; Figure S3b–d, Supporting Information). At the same time, these changes were almost completely reversed in mice with cardiac‐specific *Neu1*‐knockdown under DOX treatment (Figure 2e,f; Figure S4b–d, Supporting Information). Similarly, cardiac‐specific overexpression of *Neu1* dramatically aggravated the imbalance between antioxidant capacity and oxidative stress induced by DOX, which was evidenced by the notable increase in the content of 3‐NT, 4‐HNE, and superoxide, along with a marked decrease in the mRNA levels of *Ho1*, *Nqo1*, *Cat* and *Sod* (Figure 2g; Figure S3b,c,e,f, Supporting Information). Conversely, the cardiac‐specific knockdown of *Neu1* effectively increased the expression of antioxidant enzymes and maintained the cellular redox balance (Figure 2h; Figure S4b,c,e,f, Supporting Information).

Further investigations demonstrated that cardiac‐specific overexpression of *Neu1* profoundly suppressed the expression of key glycolytic genes, including *Ldha*, *Pfkfb1*, *Pfk1*, *Eno1*, *Pgk1*, *Pgam2*, *Pgam1*, *Gfat*, *Sord*, *Prkag2*, *Fbp2*, *Tkfc*, *Khk*, *Pmm1*, *Slc4a1*, and *Aldh1a1* (Figure 2i). This downregulation led to a pronounced inhibition of glycolytic activity, driving an abnormal glycolytic phenotype under DOX treatment. Consistently, *Neu1* overexpression dramatically downregulated both the basal glycolysis (under untreated conditions) and the compensatory glycolysis, after adding 0.5 µM rotenone/antimycin A (inhibitor of complex I and III in the electron transport chain) to completely block mitochondrial respiration in NRCMs (Figure 2j). In addition to the alterations in glycolytic flux, *Neu1* overexpression also exacerbated glycogen deposition, as evaluated by PAS staining (Figure 2k). Conversely, the cardiac‐specific knockdown of *Neu1* exhibited a distinct opposite trend of glycolytic phenotype, characterized by increased glycolysis‐related genes, promotion of glycolysis rate, and glycolysis capacity as well as the reduced deposition of glycogen (Figure 2j,l,m). Collectively, these findings suggest a promotive role of *Neu1* in chemotherapy‐evoked glucose metabolic disorders and cardiotoxicity.

### 2.3 Transcriptional Repression of *Neu1* by HIF1α Promoted the Glycolytic Phenotype

**Figure 3:**
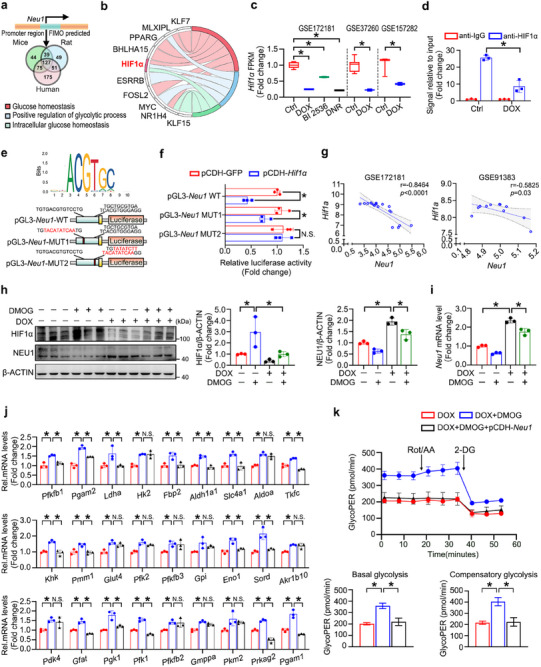
DOX Disrupts the HIF1α‐mediated Transcriptional Repression of *Neu1*, Resulting in Impaired Glycolytic Metabolism. a) Transcription factors that bind to *Neu1* were predicted using FIMO. The predictive intersection of human, mouse, and rat was screened with a condition of *p* < 0.001. b) The GO enrichment analysis was conducted to filter out the metabolism‐related factors among the predicted transcription factors. Transcription factors unrelated to glucose metabolism were not present. c) The transcriptional expression of *Hif1a* was examined in RNA‐seq data from GSE172181, GSE37260, and GSE157282. d) ChIP analyses of the *Neu1* promoters in DOX‐treated H9c2 cells were conducted using anti‐HIF1α or anti‐IgG antibodies. The precipitated chromatin DNA was examined via RT‐qPCR analyses (*n* = 3). e) The DNA binding motif of HIF1α was provided by the JASPAR database. The predicted HIF1α binding motif was then used to design specific mutations within the promoter region of the *Neu1* gene (MUT1 and MUT2). f) pCDH‐*Hif1a* or pCDH‐GFP plasmids were co‐transfected with renilla luciferase plasmid and a luciferase reporter construct containing *Neu1*‐WT promoter or mutant promoters (*Neu1*‐MUT1 or *Neu1*‐MUT2) into 293T cells. Data are presented as the relative ratio of firefly luciferase activity and renilla luciferase activity (*n* = 3). g) Correlation analysis of *Hif1a* and *Neu1* was performed in GSE172181 (*r* = −0.8464, *p* < 0.0001) and GSE91383 (*r* = −0.5825, *p* = 0.03). h,i) NRCMs were administered with 1 µM DOX and/or 1 mM DMOG for 24 h, the mRNA and protein level of NEU1 as well as HIF1α protein expression were measured by western blot and RT‐qPCR (*n* = 3). j,k) NRCMs transfected with pCDH‐*Neu1* or pCDH‐GFP were subjected to DOX treatment with or without DMOG for 24 h. The glycolysis phenotype of NRCMs was evaluated by mRNA expression levels of enzymes in the glucose metabolism (j, *n* = 3) and glycoPER (k, *n* = 5). β‐ACTIN served as an internal control and data are expressed as mean±SD. Additionally, data in (c) are presented as box‐and‐whisker plots displaying median and interquartile ranges, *p < 0.05, N.S. indicates no significance.

Given the essential role of NEU1 in DOX‐induced glucose metabolic disorders, we clarify the molecular mechanism of *Neu1* upregulation under DOX treatment. Since DOX treatment significantly elevated the mRNA level of *Neu1*, indicating that the increased NEU1 expression is probably attributed to the transcriptional regulation. Find Individual Motif Occurrences (FIMO) was employed to identify potential transcription factors binding the *Neu1* promoter region in mice, human, and rat. After rigorous filtering and analysis, a total of 127 common transcription factors were identified across species (**Figure** **3**a). Subsequently, GO enrichment analysis was performed to investigate the involvement of these 127 candidate transcription factors in glucose metabolism. Notably, 10 transcription factors exhibited significant enrichment in GO terms related to glucose homeostasis and the glycolytic process. Among these transcription factors, HIF1α was selected for further study because it uniquely exhibited significant enrichment across all three GO categories: “Glucose homeostasis”, “Positive regulation of glycolytic process” and “Intracellular glucose homeostasis” (Figure 3b). Furthermore, the decreased expression level of *Hif1α* in chemotherapy‐induced cardiomyopathy has been identified in the RNA‐seq data from GSE172181, GSE37260, and GSE157282 (Figure 3c).

Chromatin immunoprecipitation (ChIP) assays confirmed the direct binding of HIF1α to the *Neu1* promoter region, whereas treatment with DOX significantly reduced the binding affinity of HIF1α on the *Neu1* promoter (Figure 3d). Moreover, two potential binding sites for putative HIF1α‐binding motif were identified in JASPAR database (https://jaspar.genereg.net/) within the *Neu1* promoter: site 1 (−336 to −327) and site 2 (−180 to −163) (Figure 3e). To investigate this possibility, we cloned the *Neu1* promoter region, incorporating the wild‐type (*Neu1*‐WT) or mutant variants (*Neu1*‐MUT1 and *Neu1*‐MUT2), into luciferase reporter vectors. Our findings demonstrated that in 293T cells, transfection of *Neu1*‐WT followed by overexpression of *Hif1a* using the pCDH‐*Hif1α* construct led to a significant reduction in luciferase activity compared to cells transfected with pCDH‐GFP. Additionally, when *Neu1*‐MUT1 or *Neu1*‐MUT2 was co‐transfected with pCDH‐*Hif1α* into 293T cells, luciferase reporter assay results demonstrated that mutating Site2, but not Site1 in the *Neu1* promoter, resulted in a markedly greater loss of their HIF1α responsiveness (Figure 3f).

Moreover, correlation analysis displayed a significant negative correlation between *Hif1a* and *Neu1* mRNA levels (*r = −0.8464* and *r = −0.5825*, respectively) by using GSE172181 and GSE91383 datasets (Figure 3g). Subsequently, to elucidate the influence of HIF1α‐NEU1 axis in modulating glucose metabolism, NRCMs were administered with the Prolyl hydroxylase (PHD) inhibitor dimethyloxalylglycine (DMOG), which stabilizes HIF1α.^[^ ^26^ ^]^ As shown in Figure 3h‐k, DMOG not only significantly decreased the expression of NEU1 mRNA and protein level but also markedly increased glycolytic phenotype. Moreover, the overexpression of *Neu1* largely counteracted the improvement effect of DMOG on DOX‐induced glycolysis disorder, as manifested by the decreased mRNA expression of key glycolytic enzymes, lower basal glycolysis and compensatory glycolysis (Figure 3j,k).

### 2.4 Cooperativity between NRF2 and HIF1α in Regulating *Neu1* Transcript after DOX Exposure

**Figure 4:**
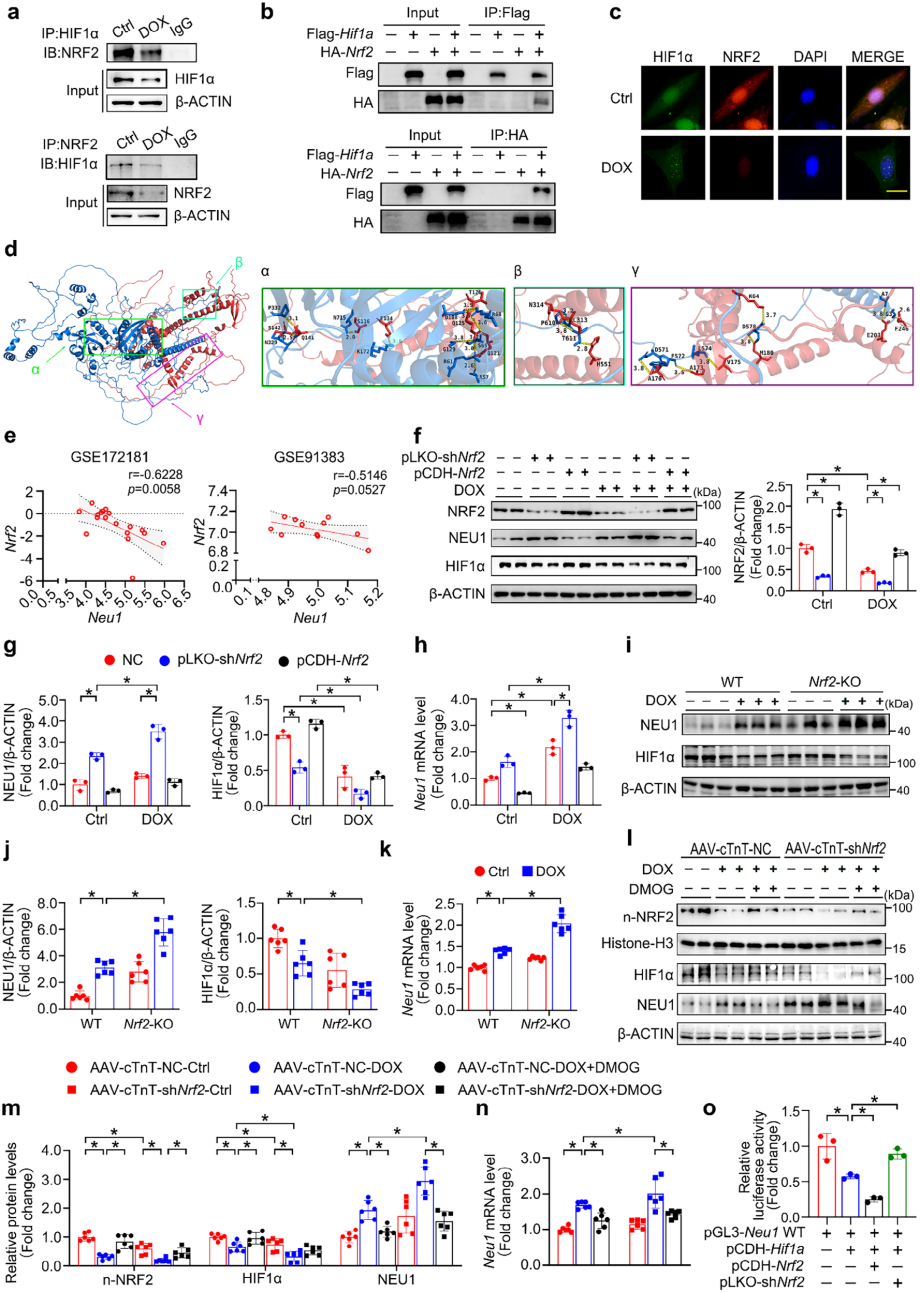
HIF1α and NRF2 Cooperatively Regulate NEU1 Expression in DIC. a) Co‐IP analysis was conducted to investigate the interaction between NRF2 and HIF1α in H9c2 cells. b) The 293T cells were co‐transfected with Flag‐*Hif1a* and HA‐*Nrf2* for exogenous overexpression, and Co‐IP assays were performed with Flag and HA antibodies. c) IF assay confirmed the co‐localization of NRF2 (Red) and HIF1α (Green) in H9c2 cells. d) Docking was performed using Z‐DOCK to predict the interaction between NRF2 (red) and HIF1α (blue). Panels α, β, and γ provide detailed views of the intermolecular hydrogen bonds between NRF2 and HIF1α. Hydrogen bonds are represented as yellow lines, with the corresponding amino acid residues indicated by their single‐letter abbreviations, and the distances measured in angstroms. e) The correlation analysis between *Nrf2* and *Neu1* was performed in GSE172181 (*r* = −0.6228, *p* = 0.0058) and GSE91383 (*r* = −0.5146, *p* = 0.0527). f–h) The mRNA and protein level of NEU1 as well as the protein level of NRF2 and HIF1α in NRCMs transfected with NC, pLKO‐sh*Nrf2*, or pCDH‐*Nrf2* plasmids under DOX treatment were examined (*n* = 3). i,j) Western blots were performed to assess NEU1 and HIF1α protein levels in WT and *Nrf2*‐KO mice after DOX treatment (*n* = 6). k) The mRNA level of *Neu1* in cardiac tissues from DOX‐treated WT and *Nrf2*‐KO mice (*n* = 6). l,m) The protein level of n‐NRF2, HIF1α and NEU1 in the heart from mice given AAV‐cTnT‐NC or AAV‐cTnT‐sh*Nrf2* and subjected to DOX and/or DMOG injection (*n* = 6). n) The RT‐qPCR was used to detect the expression of *Neu1* in cardiac tissues (*n* = 6). o) Relative luciferase activity was measured by dual‐luciferase reporter gene assay in 293T cells transfected with the *Neu1*‐WT luciferase reporter vector, alongside pCDH‐*Hif1a* and either pCDH‐*Nrf2* or pLKO‐sh*Nrf2*. Histone H3 or β‐ACTIN as an internal control. Data are expressed as mean±SD. Scale bar = 10 µm in (c), *p < 0.05.

Previous studies have suggested that the transcriptional repressor function of HIF1α always necessitates the participation of additional factors.^[^ ^27^, ^28^ ^]^ Thus, the key antioxidant factor *Nrf2*, which is known for its extensive regulatory impact on glucose metabolism homeostasis, has therefore drawn our great attention.^[^ ^29^, ^30^, ^31^ ^]^ Notably, a marked reduction of NRF2 in chemotherapy‐induced cardiotoxicity has been revealed in our previous study.^[^ ^25^, ^32^ ^]^ Thus, we intended to investigate whether NRF2 plays a role in regulating the transcriptional activity of HIF1α on *Neu1*. Through mutual Co‐IP experiments at both endogenous and exogenous levels, we found NRF2 co‐immunoprecipitated with HIF1α, confirming the presence of physical interaction between NRF2 and HIF1α (**Figure** **4**a,b). Additionally, IF staining revealed the co‐localization of NRF2 and HIF1α within the nuclei of H9c2 cells (Figure 4c). However, it is noteworthy that this close association was significantly blocked by DOX treatment (Figure 4a,c). To further characterize the molecular affinity between HIF1α and NRF2, we performed Z‐DOCK analysis, which revealed a binding energy of −33.0 kcal·mol^−1^ for the optimal docking model, along with the corresponding hydrogen bond interactions (Figure 4d).

Subsequently, we investigated the potential role of NRF2 in DOX‐mediated upregulation of *Neu1* expression. Correlation analysis conducted on public databases GSE172181 and GSE91383, which focus on chemotherapy‐induced cardiotoxicity, demonstrated a significant negative correlation between the expression levels of *Nrf2* and *Neu1*. Specifically, the correlation coefficients were found to be r = −0.6228 for GSE172181 and r = −0.5146 for GSE91383 (Figure 4e). Additionally, overexpression of *Nrf2* by pCDH plasmid containing *Nrf2* (pCDH‐*Nrf2*) resulted in a significant reduction in *Neu1* mRNA and protein levels, concomitant with a substantial elevation in HIF1α protein expression (Figure 4f–h). In contrast, silencing of *Nrf2* in cardiac tissues of both global and cardiac‐specific *Nrf2* knockout mice resulted in a remarkable reduction in HIF1α and a corresponding increase in NEU1 expression after DOX treatment (Figure 4i–n). However, DMOG treatment could effectively mitigate the effects of *Nrf2* knockdown on HIF1α and NEU1 expression (Figure 4l–n). Subsequently, to elucidate the role of NRF2 in the HIF1α‐mediated regulation of *Neu1* transcription, 293T cells harboring *Neu1*‐WT luciferase reporter constructs were co‐transfected with pCDH‐*Hif1a*, alongside either pCDH‐*Nrf2* or pLKO‐sh*Nrf2*. As shown in Figure 4o, simultaneous overexpression of *Hif1a* and *Nrf2* led to a significantly greater suppression of luciferase activity compared to *Hif1a* overexpression alone. Conversely, the knockdown of *Nrf2* partially rescued the reduction in luciferase activity caused by *Hif1a* overexpression. These results indicate that NRF2 cooperates with HIF1α to regulate *Neu1* transcription, highlighting their synergistic role in the context of chemotherapy‐induced cardiotoxicity.

Nevertheless, the binding site and the intricate mechanisms governing the interaction between HIF1α and NRF2 in the regulation of *Neu1* transcription warrant further exploration. In particular, the application of site‐directed mutagenesis could yield critical insights into the specific amino acids involved and enhance our understanding of the molecular dynamics that underlie this interaction.

### 2.5 *Nrf2*‐Mediated Glycolytic Metabolism Required for HIF1α/NEU1 Action

**Figure 5:**
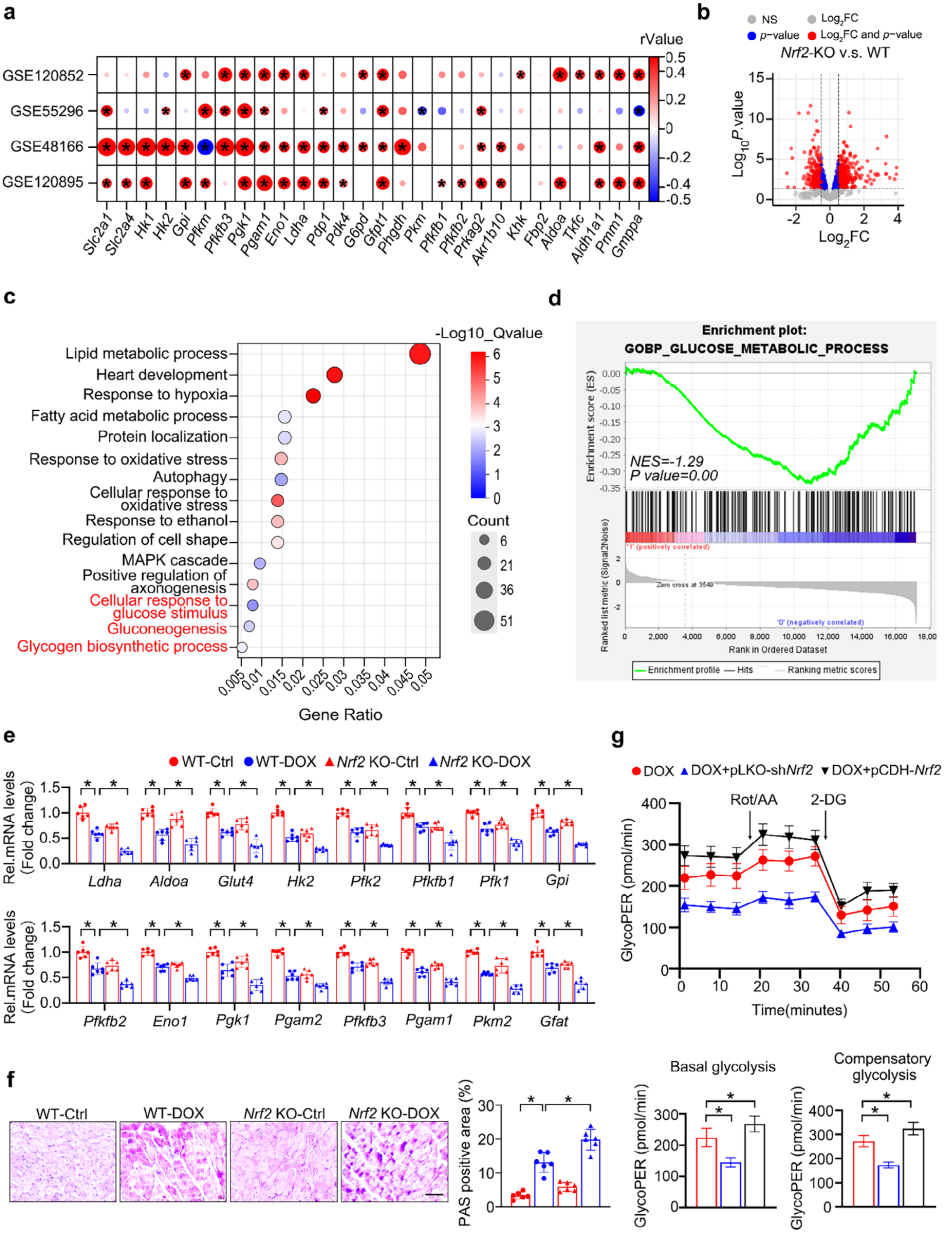
NRF2 Is a Key Regulator of Metabolic Disorder Phenotypes. a) Correlation analysis was conducted between *Nrf2* and a set of genes associated with glycolysis using publicly available transcriptomic data on heart injury obtained from the GEO database. The red color represents a positive correlation, while the blue color represents a negative correlation. The intensity of the color indicates the strength of the correlation, with white color indicating no correlation. b) The volcano plot was used to illustrate the differential gene expression between DOX‐treated WT and *Nrf2*‐KO mice. (c and d) GO c) and GSEA d) analysis was performed on the DEGs in DOX‐treated *Nrf2*‐KO mice compared with DOX‐treated WT mice. e) Relative mRNA expression levels in the heart of DOX‐treated WT and *Nrf2*‐KO mice (*n* = 6). f) Representative images and quantification of PAS staining (*n* = 6). g) The glycoPER was performed to examine the glycolytic capacities (*n* = 5). Data are expressed as mea*n* ± SD, Scale bar = 20 µm in (f), *p < 0.05.

Given that a strong correlation has been reported between the transcription factor NRF2 and glucose metabolism in tumors, adipose, and liver tissues,^[^ ^33^, ^34^, ^35^ ^]^ we analyzed several public GEO datasets to elucidate the relationship between *Nrf2* and glucose metabolism in various types of heart disease. Based on the correlation analysis in **Figure** **5**a, a statistically significant positive association was observed between *Nrf2* and lots of glycolysis enzymes in the GSE120852, GSE55296, GSE48166, and GSE120895 datasets. Subsequently, we further investigated the potential role of *Nrf2* in the process of DOX‐induced abnormal glucose metabolism. *Nrf2*‐KO mice and their littermate control WT mice were administered with DOX to induce DIC, as illustrated in Figure S5a (Supporting Information). Following treatment, RNA‐seq was performed to comprehensively characterize transcriptomic alterations in the cardiac tissue of both *Nrf2*‐KO and WT mice. Principal component analysis (PCA) distinctly separated all samples into two distinct cohorts (Figure S5b, Supporting Information), indicating a noticeable difference between the two groups. Differential gene expression analysis revealed a total of 929 differentially expressed genes (DEGs), including 524 upregulated genes and 405 down‐regulated genes when *Nrf2* was knocked out under DOX condition (Figure 5b). Notably, both GO term and gene set enrichment analysis (GSEA) revealed significant enrichment of genes involved in glucose metabolism‐related terms in the differential gene expression profile (Figure 5c,d; Figure S5c, Supporting Information). To validate the above bioinformatic analysis, PAS staining and RT‐qPCR analysis were conducted in the cardiac tissues of WT and *Nrf2*‐KO mice under DOX treatment. Indeed, treatment with DOX contributed to an enhancement in glycogen deposition and a corresponding downregulation in the expression of numerous glycolysis‐associated genes, which was further exacerbated after *Nrf2* silencing (Figure 5e,f; Figure S5d, Supporting Information).

**Figure 6:**
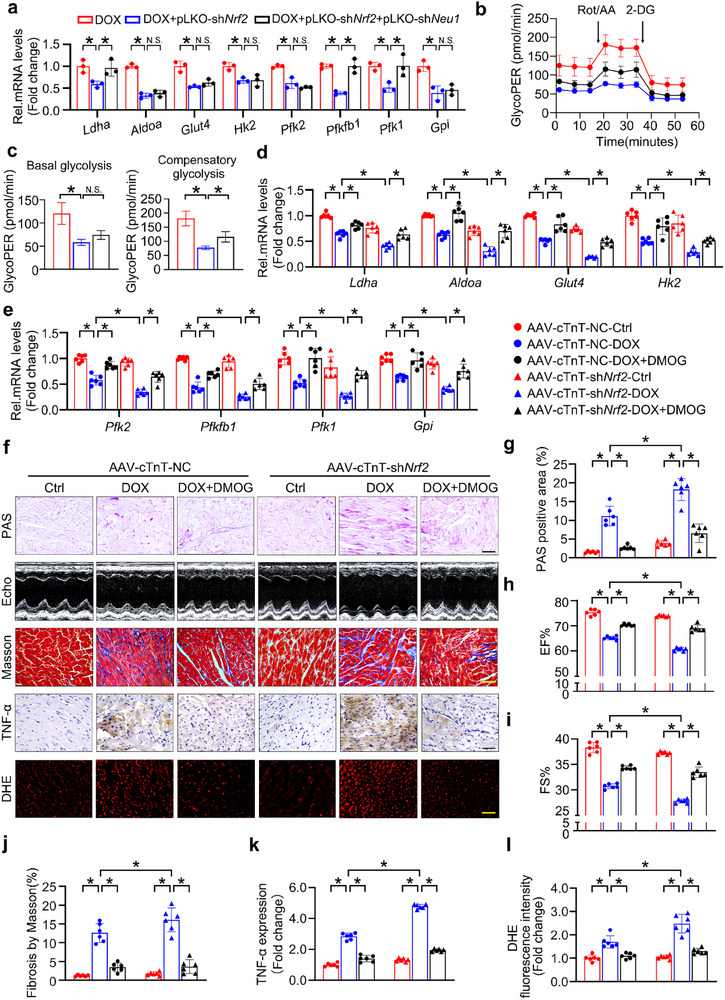
*Nrf2* Deletion Exacerbates DOX‐induced Metabolic Disorders Phenotype and Cardiac Damage through HIF1α/NEU1 Axis. a) The mRNA level of genes related to glycolysis in NRCMs transfected with pLKO‐sh*Nrf2* and/or pLKO‐sh*Neu1* under DOX treatment was tested by RT‐qPCR (*n* = 3). b,c) Quantification of basal glycolysis and compensatory glycolysis from the kinetic profiles of glycoPER (*n* = 5). d,e) Mice were administrated with AAV‐cTnT‐NC or AAV‐cTnT‐sh*Nrf2* and then subjected to DOX and/or DMOG treatment. The mRNA levels of key enzymes were quantified by RT‐qPCR (*n* = 6). f–l) Cardiac damage and glycogen deposition were detected by PAS staining, echocardiography, Masson, TNF‐α, and DHE staining (*n* = 6). Data are expressed as mean±SD, Scale bar = 20 µm in (f), *p < 0.05, N.S. indicates no significance.

Similarly, silencing of *Nrf2* in DOX‐treated NRCMs resulted in a decrease in the mRNA level of key glycolytic enzymes (e.g., *Ldha*, *Aldoa*, and *Glut4*) as well as in the basal glycolysis and compensatory glycolysis. In contrast, *Nrf2* overexpression overtly improved these destructive effects on glucose metabolism in DOX‐treated NRCMs (Figure 5g; Figure S5e, Supporting Information). Since *Nrf2* appears critical for glycolytic‐related gene expression, we next explored whether the effect of NRF2 on glucose metabolism depends on NEU1. As expected, the knockdown of *Nrf2* markedly exacerbated the DOX‐induced decrease in the mRNA level of key glycolysis‐related genes (e.g., *Ldha*, *Pfkfb1*, and *Pfk1*) as well as the impairment of basal and compensatory glycolysis. However, the detrimental effect of *Nrf2*‐knockdown on glucose metabolism was partially reversed by *Neu1*‐shRNA (**Figure** **6**a–c; Figure S6a, Supporting Information). Taken together, these results highlight that *Nrf2* deletion exacerbates metabolic disorders phenotype in a NEU1‐dependent manner.

Next, to further investigate whether HIF1α serves as a molecular bridge to link NRF2 and NEU1 during glycolysis abnormalities and DIC pathogenesis, mice were administered with AAV9‐cTnT‐*shNrf2* or AAV9‐cTnT‐NC via tail vein injection to achieve cardiomyocyte‐specific knockdown of *Nrf2* (Figure S6b, Supporting Information), and then subjected to DOX treatment with or without DMOG. As expected, the pharmacological stabilization of HIF1α effectively reversed the decrease in the mRNA levels of the key glycolysis genes as well as the increase in glycogen content induced by sh*Nrf2* (Figure 6d–g; Figure S6c, Supporting Information). Similarly, on echocardiography, DOX application induced cardiac dysfunction, indicated by a decrease in EF and FS, which was exacerbated by *Nrf2* gene knockdown. Nevertheless, DMOG administration strongly ameliorated these deficits (Figure 6f,h,i). Consistently, mice administered with AAV9‐cTnT‐sh*Nrf2* exhibited more severe structural damage, inflammatory response, and oxidative damage under DOX treatment, as evidenced by increased positive staining with Masson's trichrome, Sirius red, DHE, and IHC staining. Simultaneously, the RT‐qPCR experiment verified a significant increase in the mRNA expression levels of pro‐fibrotic (*Tgfb* and *Ctgf*) and pro‐inflammatory (*Il1b* and *Tnfa*) genes, along with a notable decrease in the expression of antioxidant genes (*Ho1*, *Nqo1*, *Cat* and *Sod*) in the DOX‐treated group with AAV9‐cTnT‐sh*Nrf2*. DMOG treatment markedly attenuated these cardiac pathological abnormalities in DOX‐treated AAV9‐cTnT‐sh*Nrf2* mice (Figure 6f,j–l; Figure S7, Supporting Information). Taken together, the stabilization of HIF1α effectively counteracted the glycolysis abnormalities and consequent cardiac injury triggered by *Nrf2* inhibition under DOX treatment.

### 2.6 NEU1 Impaired Glycolysis Phenotype through Suppression of SIRT1 Stability

**Figure 7:**
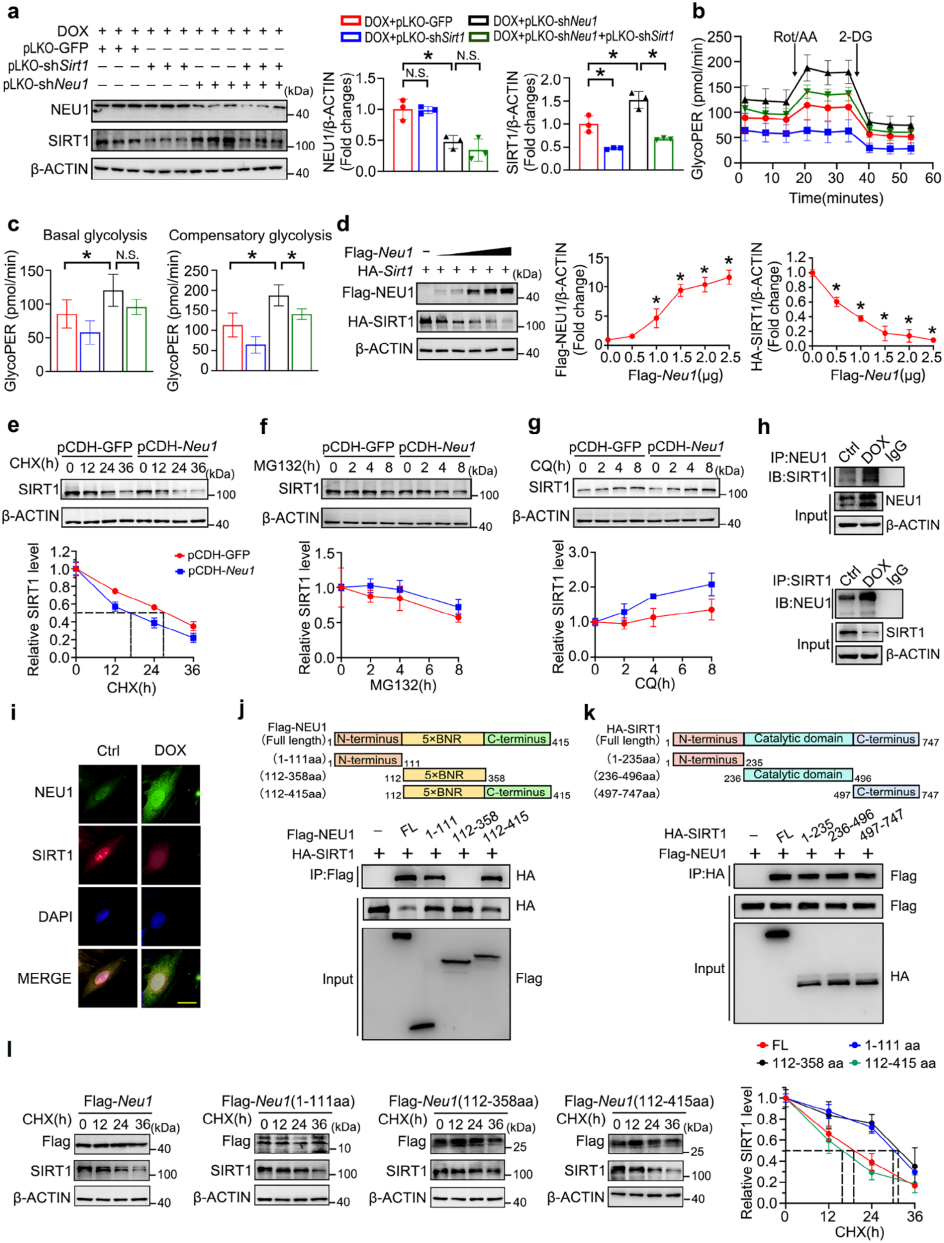
NEU1 Impairs Glycolytic Metabolism by Promoting Lysosome‐mediated Degradation of SIRT1. a) Western blot was performed to detect the expression of NEU1 and SIRT1 in NRCMs transfected with pLKO‐shNC, pLKO‐sh*Sirt1*, and/or pLKO‐sh*Neu1* followed by DOX treatment (*n* = 3). b,c) The measurement of glycoPER in NRCMs transfected with pLKO‐sh*Sirt1* and/or pLKO‐sh*Neu1* under DOX condition (*n* = 5). d) 293T cells were transfected with HA‐*Sirt1* and gradient concentrations of Flag‐*Neu1* for 48 h. The protein levels of NEU1 and SIRT1 were assessed by western blot (*n* = 3). e–g) H9c2 cells were transfected with pCDH‐GFP or pCDH‐*Neu1* for 48 h, then treated with CHX (200 µg/ml), MG132 (10 µM) or CQ (50 µM). The protein abundance of SIRT1 was measured by western blot (*n* = 3). h) The interaction between NEU1 and SIRT1 was investigated in H9c2 cells with or without DOX treatment by Co‐IP analysis. i) Representative immunofluorescence images of NEU1 (green), SIRT1 (red), and DAPI (blue) localization in H9c2 cells. j) HA‐*Sirt1*, Full‐length Flag‐*Neu1*, or truncated mutants of *Neu1* were co‐expressed in 293T cells for Co‐IP assay with anti‐Flag antibody. k) Flag‐*Neu1*, full‐length HA‐*Sirt1*, or truncated mutants of SIRT1 were co‐expressed in 293T cells for Co‐IP assay with anti‐HA antibody. l) 293T cells were co‐transfected with HA‐*Sirt1* and different truncated mutants of *Neu1* for 48 h, then the cells were treated with CHX (200ug/ml) for 0, 12, 24, or 36 h. The protein level of SIRT1 was measured by western blot (*n* = 3). β‐ACTIN as an internal control. Data are expressed as mean±SD, Scale bar = 10 µm in (i), *p < 0.05, N.S. indicates no significance.

SIRT1, an NAD^+^‐dependent histone deacetylase, has been well‐characterized for its functional role in glucose homeostasis.^[^ ^36^, ^37^, ^38^ ^]^ Our previous studies revealed that SIRT1 expression was markedly reduced in various organ injuries induced by DOX. Notably, activation of SIRT1 significantly alleviated DIC, underscoring its potential therapeutic role.^[^ ^25^, ^39^ ^]^ Therefore, we hypothesized there was a relationship between NEU1 and SIRT1 in the regulation of glycolysis. To validate this hypothesis, NRCMs were transfected with pLKO‐sh*Sirt1* and/or pLKO‐sh*Neu1* under DOX condition. The results showed that *Neu1* knockdown significantly increased the protein expression of SIRT1, while *Sirt1* knockdown had no observable impact on NEU1 protein expression (**Figure** **7**a). Importantly, the deletion of SIRT1 dramatically suppressed basal glycolysis and compensatory glycolysis, as well as the mRNA abundance related to glycolysis under DOX conditions. Moreover, the improvements in glycolytic capacities and restoration of glycolysis‐related mRNA abundance triggered by *Neu1*‐knockdown were also significantly abrogated by pLKO‐sh*Sirt1* (Figure 7b,c; Figure S8a, Supporting Information). Subsequently, to further dissect the molecular interplay between NEU1 and SIRT1, we next investigated their mechanistic relationship. Given that silencing of *Neu1* exerted negligible effects on *Sirt1* transcript levels (Figure S8b, Supporting Information), we shifted our focus to the potential impact of NEU1 on SIRT1 protein stability. Our experiments revealed that *Neu1* overexpression induced a dose‐dependent reduction in SIRT1 protein stability in 293T cells (Figure 7d). To further explore underlying mechanisms responsible for NEU1‐mediated inhibition of SIRT1 expression, the half‐life of SIRT1 protein was analyzed following treatment with protein synthesis inhibitor cycloheximide (CHX). The protein of SIRT1 underwent decay at a half‐life of ≈28 h, which was shortened to ≈17 h in cells transfected with pCDH‐*Neu1* (Figure 7e). The primary mechanisms governing protein degradation are autophagy‐lysosome and ubiquitin‐proteasome pathways.^[^ ^40^ ^]^ Upon treatment with the proteasome inhibitor (MG132) and the lysosomal inhibitor (Chloroquine, CQ), we found that CQ, rather than MG132, effectively restored SIRT1 protein expression in cells transfected with pCDH‐*Neu1* (Figure 7f,g). This observation indicates that NEU1 regulates SIRT1 degradation through the autophagy‐lysosome pathway. Furthermore, Co‐IP analyses and immunofluorescence staining revealed a high‐affinity interaction and co‐localization between NEU1 and SIRT1, following DOX stimulation (Figure 7h,i). Notably, this interaction was effectively reversed by the NEU1 inhibitor oseltamivir (OSE) (Figure S8c, Supporting Information). Additionally, the Z‐DOCK structural model successfully identified potential binding domains between NEU1 and SIRT1, with binding energies reaching up to −30.8 kcal·mol^−1^ (Figure S8d, Supporting Information).

To further characterize the specific motifs involved in the NEU1‐SIRT1 interaction, we generated a series of *Neu1* truncation mutants and integrated them with *Sirt1* constructs, followed by Co‐IP analyses. Our findings revealed that both the aa 1–111 region and aa 112–415 region of NEU1 exhibited a strong binding affinity to SIRT1, while the region spanning aa 112–358 did not demonstrate any significant interaction. These results suggest that both the N‐terminal (aa 1–111) and C‐terminal (aa 359–415) regions of NEU1 are essential for its interaction with SIRT1. Surprisingly, we found that three distinct domains of SIRT1—namely, aa 1–235, aa 236–496, and aa 497–747—could bind to full‐length NEU1, as illustrated in Figure 7j,k. To further investigate the specific domain of NEU1 responsible for mediating SIRT1 degradation, we co‐transfected various truncated mutants of NEU1 along with HA‐*Sirt1* into 293T cells, following treatment with CHX. Notably, compared with the Flag‐*Neu1* (FL), the overexpression of the 5×BNR domain (aa 112–358) and N‐terminal region (aa 1–111), rather than the 5×BNR domain combined with C‐terminal region (aa 112–415), significantly extended the half‐life of SIRT1 to ≈28 h (Figure 7l). Furthermore, to exclude the necessity of the 5×BNR domain for the degradation function of SIRT1 protein, we generated an alternative Flag‐*Neu1* overexpression plasmid that incorporated only the C‐terminal region (aa 359–415). Notably, our results demonstrated that this C‐terminal region alone was sufficient to induce the degradation of SIRT1 (Figure S8e, Supporting Information).

### 2.7 Ablation of SIRT1 Negates the Cardioprotective Effects of NRF2 During DOX Treatment

**Figure 8:**
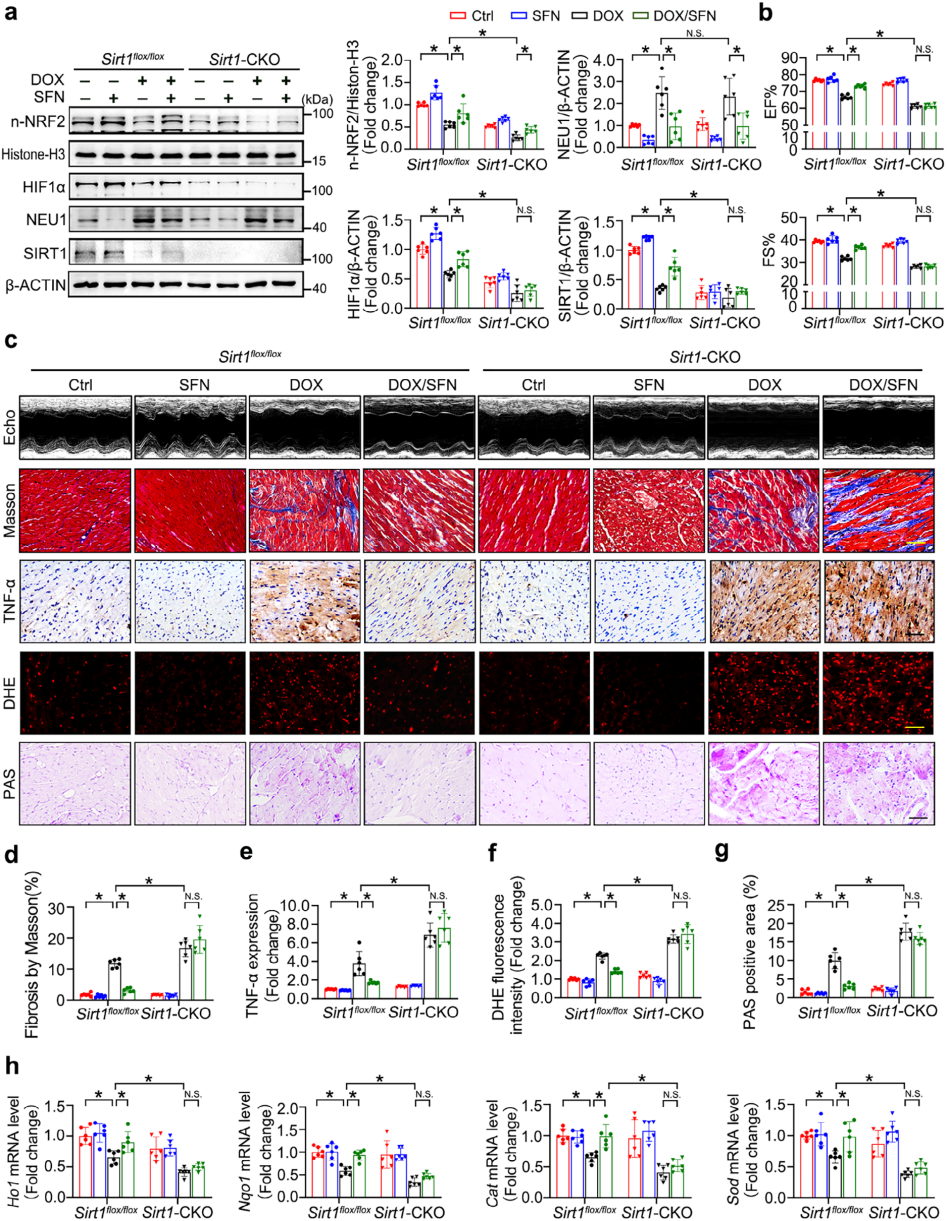
Cardiac‐specific *Sirt1* Knockout Counteracts the Protective Effects of NRF2 on Glycolytic Disorders and Cardiac Injury. a) Western blot analysis of n‐NRF2, HIF1α, SIRT1, and NEU1 protein levels in DOX and/or SFN treated *Sirt1*‐CKO mice and *Sirt1* ^flox/flox^ mice (*n* = 6). b,c) The cardiac function was accessed via echocardiography (*n* = 6). c–f) DIC was assessed by Masson, TNF‐α, and DHE staining (*n* = 6). c,g) Glycogen deposition in cardiac tissues was detected by PAS staining (*n* = 6). h) The mRNA levels of *Cat*, *Sod*, *Nqo1*, and *Ho1* were examined using RT‐qPCR (*n* = 6). Histone H3 or β‐ACTIN as an internal control. Data are expressed as mean±SD, Scale bar = 20 µm in (c), *p < 0.05, N.S. indicates no significance.

We next explored whether SIRT1 was required for the cardioprotective effects of NRF2‐mediated HIF1α‐NEU1 axis against cardiac metabolic dysfunction and structural remodeling. We first generated cardiac‐specific *Sirt1* knockout mice (*Sirt1*‐CKO) by crossing *Sirt1* ^flox/flox^ mice with Myh6‐creEsr1 mice, followed by 1‐week tamoxifen administration (Figure S9a, Supporting Information). The *Sirt1*‐CKO mice and their WT littermates were administered with DOX and/or *Nrf2* activator sulforaphane (SFN, 0.5 mg·kg^−1^) via intraperitoneal injection. Indeed, SFN administration dramatically restored DOX‐downregulated NRF2, HIF1α, and SIRT1 expression at the protein levels, concomitantly decreasing NEU1 expression in the heart. Additionally, compared with *Sirt1* ^flox/flox^ mice, *Sirt1*‐CKO significantly further inhibited the expression of NRF2 and HIF1α, but had no significant effect on the protein level of NEU1 (**Figure** **8**a). Furthermore, echocardiographic evaluation further testified that *Nrf2* activation by SFN could improve the worsened myocardial systolic dysfunction after DOX treatment (Figure 8b,c). Simultaneously, SFN‐treated mice attenuated DOX‐induced cardiac fibrosis (e.g., Masson, Sirius red, *Ctgf* and *Tgfb*), cardiac inflammatory response (e.g., TNF‐α and *Il1b*) and oxidative damage (e.g., 4‐HNE, 3‐NT, superoxide, *Ho1*, *Nqo1*, *Cat* and *Sod*) (Figure 8c–f,h; Figure S9b–f, Supporting Information). However, these protective effects of SFN against DIC were significantly impaired in *Sirt1*‐CKO mice (Figure 8b–f,h; Figure S9b–f, Supporting Information). More importantly, SFN effectively improved DOX‐impaired mRNA levels of glycolysis‐related genes, which could be almost entirely abolished by *Sirt1*‐CKO (Figure S9g, Supporting Information). Consistently, SFN‐improved glycogen deposition was also significantly dampened by *Sirt1*‐CKO following DOX stimulation (Figure 8c,g). These findings highlighted the essential role of *Sirt1* in the regulation of the NRF2 signaling pathways and glucose metabolism during DIC pathogenesis.

## 3 Discussion

Despite decades of intensive research into the pathogenesis and molecular targets of DIC, including strategies such as PEGylated liposomal DOX and adjusted dosing regimens, clinical pharmacological interventions to mitigate DIC are far from satisfactory. Emerging evidence demonstrated that glycolysis plays an extremely crucial role in DIC.^[^ ^3^, ^6^, ^7^, ^41^ ^]^ However, the exact role of glycolysis and its underlying mechanisms in chronic DIC has not been fully elucidated. To our knowledge, this study is the first to characterize the promotor role of NEU1 in DOX‐induced abnormal glucose metabolism. The major findings of the present study include the following: 1) NEU1 was remarkably upregulated in DIC. Cardiac‐specific overexpression of *Neu1* impaired glucose metabolic phenotype, whereas suppressing *Neu1* expression in cardiomyocytes effectively mitigated the adverse effects of DOX; 2) mechanistically, the upregulation of NEU1 induced by DOX was attributed to HIF1α’s transcriptional repression, which required a more sophisticated collaboration of NRF2. The pharmacological or genetic manipulation of NRF2 and HIF1α could remarkably abolish DOX‐induced upregulation of NEU1 expression and compromised glucose metabolism; and 3) NEU1 interacted with SIRT1 through its N‐terminal domain (aa 1–111) and C‐terminal domain (aa 395–415). Notably, the C‐terminal region of NEU1 promoted the lysosomal degradation of SIRT1 rather than its proteasomal degradation, thereby contributing to subsequent abnormal glycolytic phenotype and DIC.

Evidence from existing research has established that inhibition of glycolysis could independently impair cardiac contractile function and relaxation.^[^ ^7^, ^42^, ^43^ ^]^ Particularly, intermediates and products of glycolysis have been reported to participate in various vital physiological processes, including cellular growth, calcium homeostasis regulation, and sarcoplasmic reticulum function.^[^ ^7^ ^]^ DOX exposure also exerts an inhibitory effect on the transcription of key glycolytic enzymes, leading to a marked reduction in both basal and compensatory glycolysis.^[^ ^5^, ^7^, ^44^ ^]^ Consistent with these findings, bioinformatics analysis of glycolytic enzymes in multiple datasets associated with chemotherapy‐induced cardiomyopathy confirmed the inhibitory effect of DOX on glycolysis‐related gene expression in the heart. Thus, a better understanding of the molecular mechanisms governing the DOX‐triggered glycolysis is of paramount importance. Reportedly, NEU1 has been implicated in the pathogenesis of various metabolic disorders, including diabetes mellitus, obesity, and non‐alcoholic fatty liver disease, all of which are associated with dysregulated glycolysis.^[^ ^19^, ^20^, ^21^, ^22^ ^]^ Indeed, we have observed a notable upregulation of NEU1 mRNA and protein levels in both NRCMs and cardiac tissue under DOX conditions. Cardiac‐specific overexpression of *Neu1* impaired glucose metabolic phenotype after exposure to DOX, whereas suppressing *Neu1* expression in cardiomyocytes effectively mitigated these adverse effects, indicating that NEU1 may be a crucial contributor to DOX‐induced glycolytic disorder. However, the underlying mechanisms by which DOX upregulates NEU1 expression remain to be elucidated.

Considering a notable upregulation of *Neu1* mRNA levels after DOX treatment, we postulate that DOX may transcriptionally regulate NEU1 expression. We utilized the FIMO and JASPAR bioinformatics tools to screen for potential transcriptional regulators and subsequently identified HIF1α as a possible upstream regulator of NEU1 expression. A marked reduction in HIF1α protein expression was observed in both in vitro and in vivo models of DIC. Consistently, Long et al. found DOX reduced HIF1α protein levels in cardiomyocytes, and inhibiting PHD activity could effectively facilitate the stabilization of HIF1α.^[^ ^45^ ^]^ However, contrary to these findings, Wen et al. demonstrated that ROS triggered by DOX can inhibit PHD activity, thereby attenuating the degradation of HIF1α in cardiac tissue.^[^ ^46^ ^]^ These discrepancies may be attributed to variations in cell types, drug exposure duration, and dosages of DOX, as well as differences in the experimental systems employed. Generally, HIF1α’s transcriptional activation or repression of specific gene promoters varies depending on cell type and gene promoter.^[^ ^47^, ^48^, ^49^, ^50^ ^]^ In the present study, we employed ChIP‐PCR and luciferase reporter assays to demonstrate that HIF1α predominantly binds to the −180 to −163 region in the promoter of *Neu1*, thereby regulating its transcriptional repression. While HIF1α is well‐recognized as a potent transcriptional activator of glycolysis in cardiac pathogenesis, the upstream triggers for its activation remain unclear. In the present study, we revealed that NRF2 may function as a molecular bridge, linking HIF1α and NEU1 to facilitate glucose metabolism, particularly during the glycolytic process. However, the exact regulatory mechanisms between NRF2 and HIF1α in chemotherapy‐based glycolytic phenotype and cardiotoxicity still need to be addressed.

Additionally, the molecular mechanisms by which NEU1 is implicated in glycolytic processes within the heart remain unspecified. NEU1 has been reported to aggravate mitochondrial dysfunction by inhibiting SIRT1. As a deacetylase, SIRT1 regulates the activity of various metabolic enzymes via deacetylation modification, thereby modulating glucose and lipid metabolism.^[^ ^51^, ^52^ ^]^ Therefore, we hypothesized that NEU1 may regulate glucose metabolism by inhibiting SIRT1. It was found that under DOX stimulation, the knockdown of the *Neu1* gene displayed an improved glycolysis phenotype, which could be significantly rescued by *Sirt1* knockdown. Further investigations demonstrate that NEU1 suppresses the stability of SIRT1 mainly through modulation of its protein degradation. The autophagy‐lysosome system and the ubiquitin‐proteasome system are the two predominant systems for intracellular proteolysis in eukaryotic cells.^[^ ^40^ ^]^ In our study, we found that NEU1‐mediated degradation of SIRT1 was completely inhibited by an inhibitor of the lysosome (CQ) rather than an inhibitor of the proteasome (MG132), which suggests that NEU1 regulates SIRT1 expression through the lysosomal degradation pathway. Subsequently, we established that NEU1 interacts with SIRT1 via both the N‐terminal (aa 1–111) and C‐terminal (aa 359–415). Moreover, the C‐terminal region contains a COOH‐terminal tyrosine phosphorylation motif that plays a pivotal role in directing plasma membrane proteins to endosomes.^[^ ^53^ ^]^ In the present study, we also found that this region is responsible for promoting the degradation of SIRT1. Furthermore, the lysosomal degradation of SIRT1 appears to hinge on its phosphorylation status.^[^ ^54^ ^]^ Consequently, a critical question arises as to whether the NEU1‐mediated lysosomal degradation of SIRT1 is influenced by the phosphorylation status of SIRT1. This regulatory mechanism may parallel findings in the renal fibrosis model, where NEU1 has been observed to affect the ALK5 phosphorylation.^[^ ^55^ ^]^

Last but not least, aberrant NEU1 expression has been implicated in numerous malignancies and is positively associated with poor prognosis (Figure S10a–c, Supporting Information).^[^ ^56^, ^57^, ^58^ ^]^ Considering that an ideal therapeutic target for DIC should not only mitigate cardiac damage but also impede cancer progression, we further investigated the role of NEU1 in cancer chemotherapy. In nude mice bearing PC‐3 human prostate cancer xenografts, DOX treatment significantly downregulated NEU1 expression while concomitantly increasing SIRT1 protein levels in tumor lysates (Figure S10d, Supporting Information). Moreover, we further revealed that NEU1 inhibitor OSE demonstrated a dose‐dependent inhibitory effect on PC‐3 cells proliferation (Figure S10e, Supporting Information). Notably, the combination of DOX and OSE synergistically amplified anti‐tumor effects, evidenced by a significant reduction in proliferating cells, an increase in TUNEL‐positive cells, and the upregulation of pro‐apoptotic markers (Figure S10f–j, Supporting Information). These findings highlight the potential of OSE as a synergistic agent in enhancing the efficacy of DOX, offering a promising therapeutic strategy for improving outcomes in cancer treatment.

## 4 Conclusion

**Figure 9:**
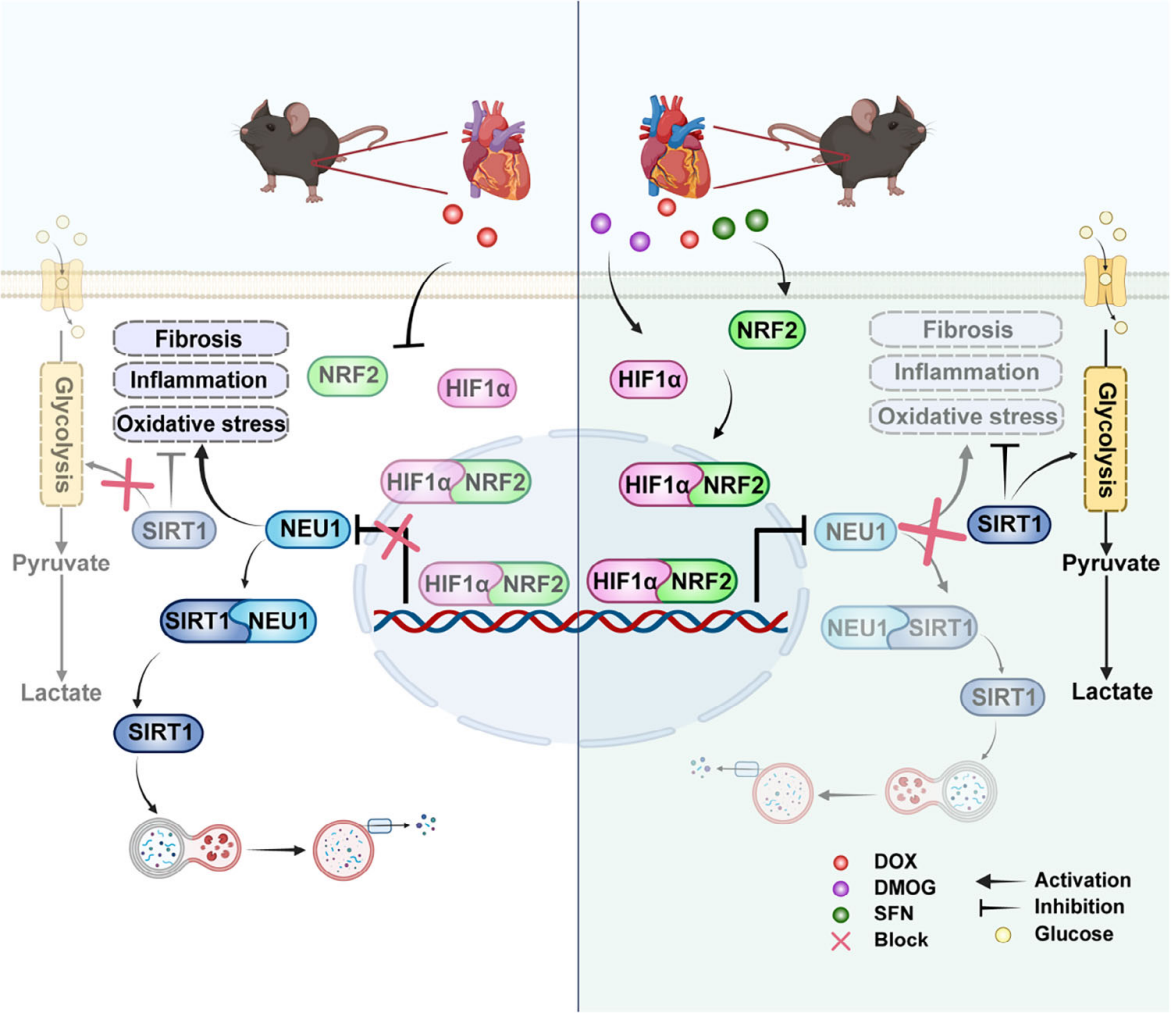
Schematic Illustration of DOX Exacerbates Glycolytic Dysregulation and Cardiotoxicity by Augmenting NEU1‐mediated SIRT1 Degradation via NRF2/HIF1α Signaling Suppression.

In this study, we unveiled a previously unrecognized role of NEU1 as a master manipulator of defective glycolysis, and cardiotoxicity under chemotherapeutic stress conditions. Our mechanistic investigations demonstrated that DOX upregulates NEU1 expression through transcriptional repression mediated by HIF1α on the *Neu1* promoter, necessitating a complex interplay with NRF2. Furthermore, the interaction between the C‐terminal region of NEU1 (aa 359–415) and SIRT1 has been identified as a critical factor that predisposes SIRT1 to destabilization via the lysosomal degradation pathway. This interplay ultimately exacerbates the pathophysiology of DIC by perturbing the metabolic network (**Figure** **9**). Therefore, targeting aberrant NEU1 expression may represent a promising clinical strategy for managing metabolic adaptability in the heart, potentially improving clinical outcomes for cancer patients undergoing chemotherapy.

## 5 Experimental Section

Detailed descriptions of the animal models and methodologies could be found in the supporting information.

### Animals Models

In this study, it was conducted five distinct sets of animal experiments: 1) to elucidate the role of NEU1 in the dysregulation of glycolysis and DIC; 2) to assess the function of NRF2 in glucose metabolic pathways; 3) to investigate whether HIF1α acts as a crucial intermediary linking NRF2 and NEU1 in the context of glycolytic dysregulation and DIC pathogenesis; 4) to evaluate the impact of SIRT1 on DIC and metabolic disturbances; and 5) to explore the role of NEU1 in tumor‐bearing mice undergoing anthracyclines chemotherapy. All animal experimental procedures were approved by the Institutional Animal Welfare and Use Committee of Shandong University (permit number: 2023‐D‐004).

### Echocardiography

Transthoracic echocardiography (Vevo 2100, Visual Sonics, Toronto, ON, Canada) was performed on mice anaesthetized with 1–2% isoflurane (Cat# R510‐22 ‐ 8, RWD Life Science Co., Ltd., China), maintaining heart rate, respiratory rate, and body temperature within standard limits as previously reported.^[^ ^25^, ^32^ ^]^ The left ventricle was assessed in parasternal long‐axis views. After measurements, values such as the EF and FS were calculated according to guidelines.

### ChIP Assay

ChIP was performed to explore HIF1α occupancy at the *Neu1* promoter in H9c2 cells according to the manufacturer's instructions (Cat# 9002, Cell Signaling Technology, USA). Briefly, H9c2 cells were treated with or without DOX (1 µM, Cat# A3966, APExBIO Technology LLC, USA) for 24 h. Cells were then crosslinked with 1% formalin for 10 min and lysed, the chromatin was harvested for fragmented. The digested cross‐linked chromatin was incubated with anti‐HIF1α antibodies (Cat# 20960‐1‐AP, Proteintech, China) or non‐immune IgG overnight at 4 °C with rotation. Subsequently, DNA was purified and used for subsequent PCR analysis.

### Seahorse XFe96 Extracellular Flux Analysis

Glycolytic proton efflux rate (glycoPER) was detected using the Seahorse XF96 Extracellular Flux Analyzer (Agilent Technologies, Germany) according to the manufacturer's instructions (Cat# 103344‐100, Agilent Technologies, Germany). Briefly, NRCMs were plated into the XF96 cell culture plates. After 48 h of incubation, NRCMs were transfected with various plasmids for 24 h, following by treatment with DOX and/or DMOG (Cat# HY‐15893, MedChemExpress, USA). Subsequently, the Glycolytic Rate assay was conducted according to the instructions, and the Seahorse XF Glycolytic Rate Assay Report Generator was utilized to calculate the glycoPER.

### Statistical Analysis

Unless specified otherwise, all the experimental data were expressed as the mea*n* ± standard deviation (mean±SD). The Shapiro‐Wilk normality test was employed to assess the normal distribution of the data before analysis. The normally distributed data were examined by unpaired Student's *t*‐test or one‐way ANOVA followed by Bonferroni multiple comparison tests. For data involving two independent variables, two‐way ANOVA with Bonferroni's multiple comparison tests was utilized. Nonparametric statistical tests were applied when data did not meet normality assumptions. The unpaired two‐tailed Mann‐Whitney U test was used for comparison between two groups and the Kruskal‐Wallis test with the Dunn multiple comparisons post hoc test was used for comparison between multiple groups. *p* < 0.05 was considered statistically significant. Data were analyzed using Prism software (version 9. 0) and R (Version 4.4.1).

## Conflict of Interest

The authors declare no conflict of interest.

## Author Contributions

J.L.G. conceptualized the study and was responsible for funding acquisition, resources and supervision; T.G. performed experiments and drafted the manuscript. J.W., X.H.Z. and Q.B.L. were responsible for the methodology; G.P.L., J.H.L. and M.R.L. were responsible for the investigation. D.M.Z., X.Y.T., X.G. and S.X.L. were responsible for visualization. Y.F.T. and T.Z. were responsible for project administration.

## Supporting information



Supporting Information

## Data Availability

The data that support the findings of this study are available from the corresponding author upon reasonable request.
