# Supplementary material for: Neuraminidase 1 Exacerbated Glycolytic Dysregulation and Cardiotoxicity by Destabilizing SIRT1 through Interactions with NRF2 and HIF1α
Source: Adv Sci (Weinh). 2025 May 24;12(25):2414504. doi: 10.1002/advs.202414504 (PMC12224949; doi:10.1002/advs.202414504)
Supplement: Supplementary file 1 — Supporting Information [file ADVS-12-2414504-s001.docx]

**Neuraminidase 1 Exacerbated Glycolytic Dysregulation and Cardiotoxicity by Destabilizing SIRT1 through Interactions with NRF2 and HIF1α**

Ting Gao^1^, Yufeng Tang^2^, Tao Zeng^3^, Jie Wang^1^, Xiaohui Zhang^1^, Qingbo Liu^1^, Xun Guan^1^, Xinyu Tang^1^, Guangping Lu^1^, Jiahao Li^1^, Mingrui Liu^1^, Dongmei Zhang^1^, Sixuan Lv^1^, Junlian Gu^1^*

^1^ School of Nursing and Rehabilitation, Cheeloo College of Medicine, Shandong University, Jinan, Shandong, 250012, China

^2^ Department of Orthopedic Surgery, The First Affiliated Hospital of Shandong First Medical University, Jinan, Shandong, 250014, China

^3^ School of Public Health, Cheeloo College of Medicine, Shandong University, Jinan, Shandong, 250012, China

*** Corresponding author:** E-mail: junlian_gu@sdu.edu.cn

**Address correspondence to:**

Dr. Junlian Gu, School of Nursing and Rehabilitation, Cheeloo College of Medicine, Shandong University. Address: No. 44, Wenhua West Road, Jinan, Shandong, 250012, China.

**Running title:** NEU1 in glycolysis disorder and cardiotoxicity

**This PDF file includes:**

Experimental Section

Figures. S1 to S10

1. **Experimental Section**

**1.1. Animals Models**

Animals were maintained in the Model Animal Research Center at Shandong University, Jinan, China. Male mice aged 8-10 weeks and weighing 23-25g were used in this study. Mice had free access to a standard rodent diet and tap water and were housed in a temperature-, light- and humidity-controlled specific pathogen-free facility. The researchers were blinded to the genotypes of the mice during the experiment and data evaluation. All the mice were included for analysis. No exclusion criteria were predetermined, and no animals were excluded.

Five sets of animal models were applied. To investigate the effect of NEU1 on glycolysis disorder and DIC, AAV9 vectors carrying the cardiac troponin T (cTNT) promoter driving the expression of enhanced green fluorescent protein (GFP) (AAV9-cTNT-GFP) and shRNA targeting *Neu1* (AAV9-cTNT-GFP-sh*Neu1*) or the *Neu1* gene were constructed and purchased from OBiO Technology (Shanghai, China). C57BL/6J mice were acquired from Vital River Laboratories (Beijing, China) and randomly divided into four groups (6 mice per group): AAV-cTnT-NC-DOX group, AAV-cTnT-sh*Neu1*-DOX group, AAV-cTnT-GFP-DOX group and AAV-cTnT-*Neu1*-DOX group. Then, mice were given different types of AAV vectors by tail vein injection at a dose of 5×10^11^ vector genomes (vg)/mouse according to the different experimental groups. After 3 weeks of recovery, DOX (5 mg/kg, Cat# HY-15142, MedChemExpress, USA) or the same volume of vehicle (saline) was injected intraperitoneally once a week for 3 weeks (15 mg/kg cumulative dose)^[1]^.

Wild-type (WT) and *Nrf2* knockout (*Nrf2*-KO) mice on a C57BL/6J background were generously provided by Dr. Tao Zeng (School of Public Health, Shandong University). Mice of the same genotype were randomly assigned to the Control group (Ctrl) and DOX-treated group (6 mice per group). Then, to establish a chronic cardiomyopathy mouse model, DOX was injected intraperitoneally, as described above.

To induce cardiomyocyte-specific *Nrf2* knockdown, AAV9-cTNT-NC and AAV9-cTNT-GFP-sh*Nrf2* were purchased from OBiO Technology (Shanghai, China). Mice were randomly divided into six groups (6 mice per group): AAV-cTnT-NC-Ctrl group, AAV-cTnT-NC-DOX group, AAV-cTnT-NC-DOX+DMOG group, AAV-cTnT-sh*Nrf2*-Ctrl group*,* AAV-cTnT-sh*Nrf2*-DOX group and AAV-cTnT-sh*Nrf2*-DOX+DMOG group. Then, the AAV infection was performed as described previously. After 3 weeks of recovery, DOX was injected intraperitoneally to induce chronic cardiotoxicity; meanwhile, DMOG (8 mg/injection) or the same volume of vehicle (saline) was intraperitoneally injected every other day for 3 weeks to stabilize HIF1α^[2, 3, 4, 5]^.

To verify the function of SIRT1 on DIC and metabolism disorder, cardiac-specific *Sirt1* knockout (*Sirt1*^flox/flox^ *Myh6*-cre/*Esr1*, *Sirt1*-CKO) mice on the C57BL/6J background were generated by crossing *Sirt1*^flox/flox^ mice with *Myh6-*cre/*Esr1* mice (Cyagen Biosciences Inc., Suzhou, China), followed by a week of tamoxifen administration. Two weeks after the last tamoxifen injection, *Sirt1*-CKO mice and *Sirt1*^flox/flox^ mice were randomly assigned to four groups (6 mice per group); Ctrl group, SFN group, DOX group as well as DOX and SFN co-treatment group (DOX/SFN). The DOX-induced chronic cardiomyopathy model was established as described above. At the same time, to activate NRF2, 0.5mg/kg of SFN (Cat# HY-13755, MedChemExpress, China) or the same volume of vehicle (saline) was administered intraperitoneally, 5 days a week, for three weeks^[6]^.

To explore the role of NEU1 in anthracyclines chemotherapy, 12 male BALB/c nude mice (nu/nu, 4–5 weeks of age) were purchased from Vital River Laboratories (Beijing, China) for tumorigenesis experiment. Specifically, to establish subcutaneous xenograft models, PC-3 cells (5×10^6^ in 100 μl) were injected into the right subcutaneous area of mice. The volume of xenografts was measured every other day and calculated using the formula: volume (mm^3^) = (length·width^2^)/2. Once the subcutaneous tumor volume reached approximately 100 mm³, mice were randomly assigned to receive either normal saline or DOX (5 mg/kg) via intraperitoneal injection once a week for three weeks.

**1.2. Cell Culture and Treatments**

NRCMs were isolated from 1 to 3-day-old Sprague-Dawley rats, as described previously^[7]^. Briefly, after sacrificing the rat, hearts were removed, minced, and washed with cold Hanks’ buffered saline solution until the liquid became clear. Then, the cardiac tissue was digested with collagenase II (Cat# V900892, Sigma-Aldrich, St. Louis, MO, USA) for 2h at 37% with permanent gentle shaking to isolate cells. The cell suspension was centrifuged and filtered through a 70-µm cell strainer to remove tissue debris. Next, plating resuspended cells in 100-mm culture dishes with 10% new-born calf serum, 6% horse serum (Kang Yuan biology, Zibo, China), 1% 5-Bromo-2′-deoxyuridine (Sigma-Aldrich), 1% antibiotics (10KU/ml penicillin and 10 mg/ml streptomycin) (Gibco, Grand Island, NY, USA) for 1h to allow cardiac fibroblasts adhere. Then, the NRCMs will be retrieved for the following analysis.

H9c2 cells (an embryonic rat myocardium-derived cell line) and 293T cells purchased from the American Type Culture Collection (ATCC, VA, USA) were cultured in 4.5 g/L glucose Dulbecco’s modified Eagle’s medium (Cat# CM10013, Macgene, Beijing, China) supplemented with 10% fetal bovine serum (Cat# FSP500, Excell Bio, China) and 1% antibiotics. Human prostate carcinoma cell line PC-3 cells obtained from HyCyte (Suzhou, China), was cultured in Ham’s F12K medium (Cat# CM10025, Macgene, Beijing, China) supplemented with 10% fetal bovine serum and 1% antibiotics.

NRCMs or H9c2 cells were selected to treat with DOX (1μM) for 24 h to establish DIC in vitro^[1, 7]^. To explore the function of NRF2 and HIF1α in DIC, SFN (10μM) or DMOG (1mM) was used to activate NRF2 or stabilize HIF1α in vitro^[6, 8, 9]^.

**1.3. Plasmids and Virus**

The plasmids pcDNA3.1-HA-*Sirt1* and pcDNA3.1-Flag-*Neu1* were generated by cloning the PCR-amplified human *Sirt1,* or human *Neu1* gene into pcDNA3.1-HA vector or pcDNA3.1-Flag vector. Truncated constructs of *Sirt1 or Neu1* were generated by ligating PCR–amplified cDNA fragments encoding the specified domains into the pcDNA3.1-HA vector or pcDNA3.1-Flag vector.

shRNA constructs were generated by inserting shRNA targeting sequences into pLKO.1-GFP vector. Plasmids for rat *Nrf2*, human *Nrf2*, rat *Neu1,* and human *Hif1a* were generated in the vectors of pCDH-CMV-EF1-GFP by molecular cloning. According to the manufacturer’s protocol, H9c2 cells and 293T cells were transfected using INVI DNA RNA Transfection Reagent (Cat# IV1216075, Invigentech, USA).

**1.4. Histological and Cellular Staining**

Fresh mice hearts were fixed in 10% formalin at room temperature, dehydrated, embedded in paraffin, and sectioned into 4-µm thickness. Masson’s trichrome staining (Cat# G1006, Servicebio Technology, China) and Sirius red staining (Leagene Biotechnology, China) were performed to evaluate cardiac collagen deposition. To detect ROS production, dihydroethidium (DHE) staining (Cat# S0063, Beyotime Biotechnology, China) was conducted. IHC staining was performed by using anti-tumor necrosis factor-α (TNF-α, 1:200, Cat# ab6671, Abcam, USA), anti-3-nitrotyrosine (3-NT, 1:300, Cat# AB5411, Millipore, USA) and anti-4-hydroxynonenal (4-HNE, 1:300, Cat# ab46545, Abcam, USA) antibody as previously^[1, 6, 7, 9, 10, 11]^. The IF staining of NRF2, HIF1α, SIRT1 and NEU1 in H9c2 cells using anti-NRF2 (1:200, Cat# ab31163, Abcam, USA), anti-HIF1α (1:200, Cat# BF0593, Affinity Biosciences, China), anti-NEU1 (1:200, Cat# 67032-1-Ig, Proteintech, China) and anti-SIRT1 (1:200, Cat# A00018-1, BOSTER, China). Additionally, the terminal deoxynucleotidyl transferase dUTP nick-end labeling (TUNEL) staining (Cat# 12156792910, Roche, Meylan, France), and Edu staining (Cat# C0078S, Beyotime Biotechnology, China) was utilized to assess apoptosis and cell proliferation in PC-3 cells.

All the sections and cells were evaluated by the biological microscope (Nikon, Tokyo, Japan) or fluorescence microscope (Nikon, Tokyo, Japan) and quantified using Image J software.

**1.5. Quantitative Real-Time Reverse Transcription Polymerase Chain Reaction (RT-qPCR)**

Total RNA was extracted from heart tissue or cells using a Trizol reagent (Cat# AC0101-A, SparkJade, China). Only RNA samples with a purity (OD 260 /OD 280) ratio of 1.8~2.0 were utilized for subsequent cDNA synthesis using the cDNA Synthesis Kit (Cat# 11141ES60, Yeasen, China). RT-qPCR was conducted with Hieff qPCR-SYBR Green master mix (Cat# 11201ES08, Yeasen, China) in a Roche LightCycler 480 (Roche, Germany).

**1.6. Western Blot Analysis**

Western blotting was done as previously described^[1, 6, 7, 9, 10, 11]^. Membranes were incubated overnight at 4℃ with the following primary antibodies: anti-SIRT1 (1:1000, Cat# 9475, Cell Signaling Technology, USA), anti-NRF2, anti-NEU1, anti-β-ACTIN (1:1000, Cat# 66009-1-Ig, Proteintech, China), anti-HIF1α, anti-Flag (1:5000, Cat# 66008-4-Ig, Proteintech, China), anti-HA (1:5000, Cat# 51064-2-AP, Proteintech, China), anti-Histone H3 (1:3000, Cat# BF9211, Affinity Biosciences, China), anti-BAX (1:5000, Cat# 50599-2-Ig, Proteintech, China), anti-Caspase 9 (1:1000, Cat# 10380-1-AP, Proteintech, China), anti-Cytochrome c (1:1000, Cat# 10993-1-AP, Proteintech, China), anti-BCL-2 (1:3000, Cat# BF9103, Affinity Biosciences, China) , and anti-Cleaved Caspase-3 (1:1000, Cat# 9664, Cell Signaling Technology, USA). The secondary antibodies (1:8000, Cat# 111-035-003 and Cat# 115-035-003, Jakson, USA) were incubated at room temperature for 1 h. The signal intensities were visualized using an Enhanced chemiluminescence (ECL) detection kit (Cat# BL520B, Biosharp, China) and analyzed by using Image J software.

**1.7. Immunoprecipitation Assay**

Immunoprecipitation (IP) assays were performed as described previously^[1, 7]^. H9c2 cells were harvested and lysed using an IP buffer containing protease and phosphatase inhibitors (Cat# P0013, Beyotime Biotechnology, China). The lysates were incubated at 4°C with antibodies against Flag, HA, HIF1α, NRF2, SIRT1, NEU1, or normal IgG for 2 h. Subsequently, protein A/G PLUS-agarose beads (30 μl) (Cat# sc-2003, Santa Cruz Biotechnology, USA) were added to the lysates at 4 °C overnight. After washing and centrifugation, the immunocomplexes were analyzed using western blotting.

**1.8. Dual-luciferase Reporter Assay**

A dual-luciferase reporter gene assay (Cat# E1910, Promega, USA) was adopted as previously described^[6, 10]^. Firstly, pGL3-*Neu1*-promoter-WT or pGL3-*Neu1*-promoter-mutant (MUT) was conducted by subcloning the *Neu1* promoter region, with or without mutated HIF1α binding sites (-336 to -327 and -180 to -163) into the pGL3-basic luciferase reporter plasmid vector. Then, pCDH-CMV-EF1-GFP-*Hif1a* and renilla luciferase plasmid were co-transfected with pGL3-*Neu1*-WT, pGL3-*Neu1*-promoter-MUT1 or pGL3-*Neu1*-promoter-MUT2 reporter plasmids into 293T cells. After 48h of transfection, cells were lysed, and the supernatant was collected for further analysis.

**1.9. Protein-protein Docking**

The predicted protein structure of NRF2, HIF1α, NEU1 and SIRT1 were retrieved from the AlphaFold Protein Structure Database (https://alphafold.ebi.ac.uk/). Then, the protein–protein docking was conducted using ZDOCK (http://zdock.umassmed.edu/) for rigid protein docking analysis. Subsequently, the amino acids and their molecules involved in hydrogen bond formation in the predicted docking models generated by the ZDOCK server were evaluated using PDBePISA (https://www.ebi.ac.uk/msd-srv/prot_int/). Finally, visual modeling of this predicted interactions was performed by PyMOL software.

**1.10. CCK8 assay**

The CCK8 assay was conducted according to the manufacturer’s protocol (Cat# K1018, APExBIO Technology, Houston, USA). Briefly, PC-3 cells were seeded into 96-well plates at a density of 5×10³ cells per well and allowed to adhere for 24h. Following this, various concentrations of DOX and OSE was applied to stimuli PC-3 cells for 24h. After then, 10μl CCK8 solution was added to each well, and the cells were incubated at 37°C under 5% CO_2_ for 1h. The optical density (OD) at a wavelength of 450 nm for each well were measured using a microplate reader. Cell viability was calculated according to the following equation: (drug-supplemented OD-blank control OD)/ (normal control OD-blank control OD) ×100%.

**
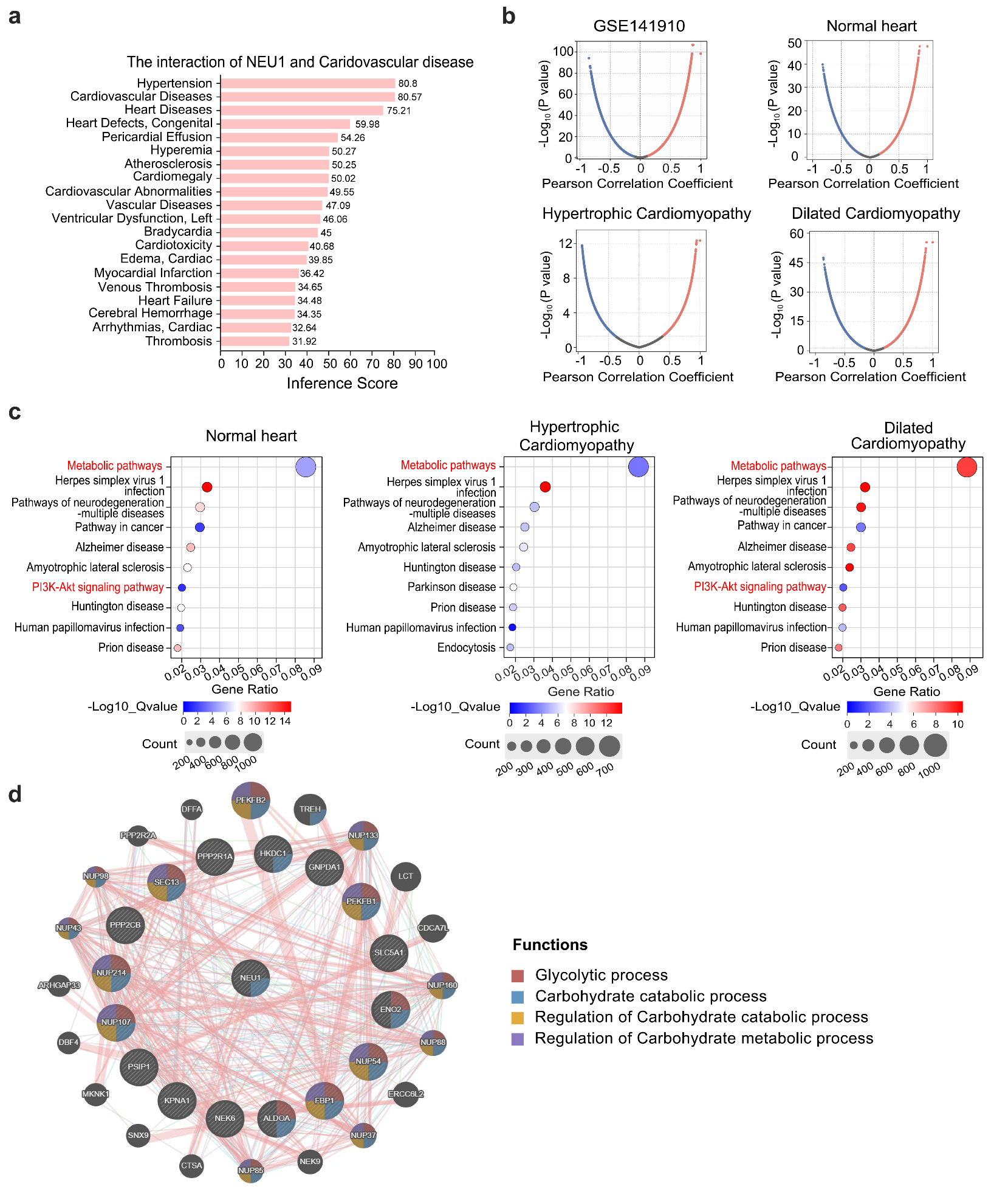
**

**Figure S1. NEU1 Plays a Critical Role in Metabolism-related Disease.** a) Interactions between the *Neu1* and cardiovascular disease based on the CTD. b) The volcano map displayed the co-expression genes associated with NEU1 expression in NCM, DCM and HCM patients of GSE141910. c) Enrichment analysis of KEGG terms was conducted for *Neu1* co-expression genes in the NCM, DCM and HCM patients of GSE141910. d) The protein-protein interactions (PPI) between NEU1 and genes related to glycolysis were analyzed using the GeneMANIA database.


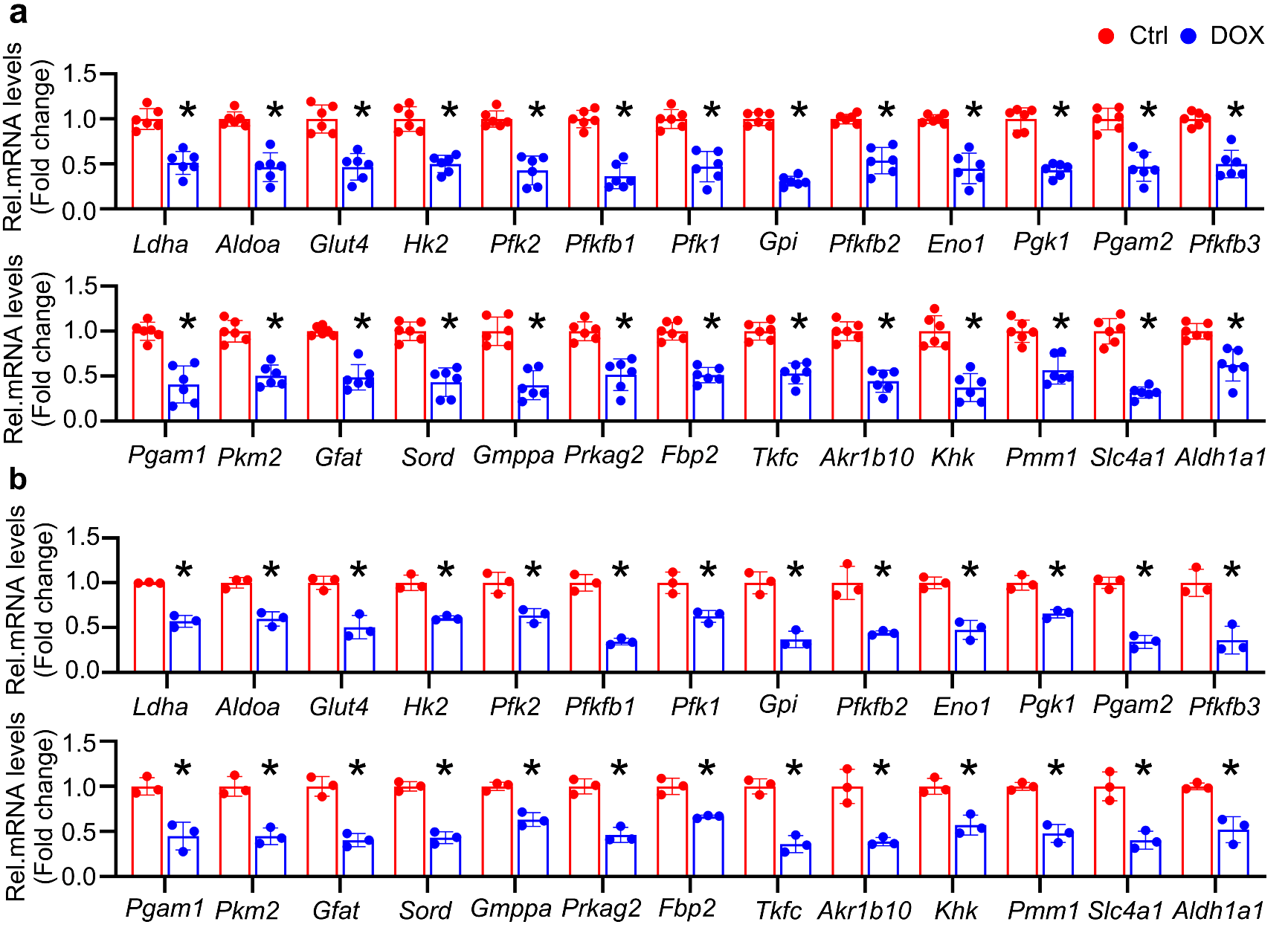


**Figure S2. DOX Disrupts Glucose Metabolism in the Heart.** The mRNA levels of multiple glucose metabolic enzymes were quantified by RT-qPCR in (a) cardiac tissues (n=6) and (b) NRCMs (n=3). Data are expressed as mean±SD, **p* < 0.05.


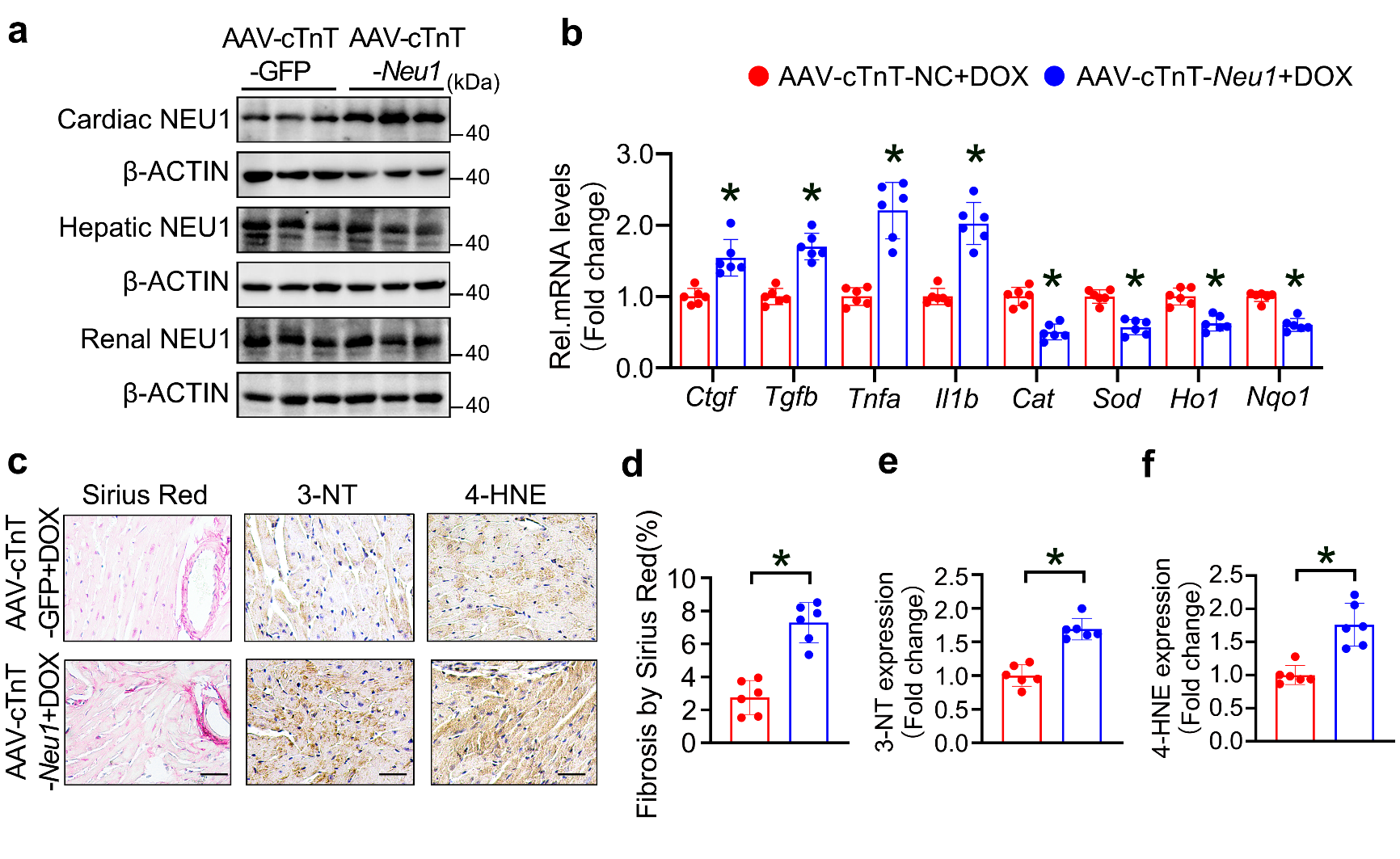
**Figure S3. Cardiac-specific *Neu1* Overexpression Markedly Exacerbated DOX-induced Cardiac Damage.** a) Representative western blot images of NEU1 in various organs of mice given AAV-cTnT-GFP or AAV-cTnT-*Neu1*. b) Relative mRNA levels of *Ctgf*, *Tgfb*, *Tnfa,* *Il1b, Cat*, *Sod*, *Ho1* and *Nqo1* (n=6). c-f) The representative image and quantitation of Sirius red, 3-NT and 4-HNE staining in cardiac tissue (n=6). β-ACTIN as an internal control. Data are expressed as mean±SD, Scale bar=20μm in (c), **p* < 0.05.

**
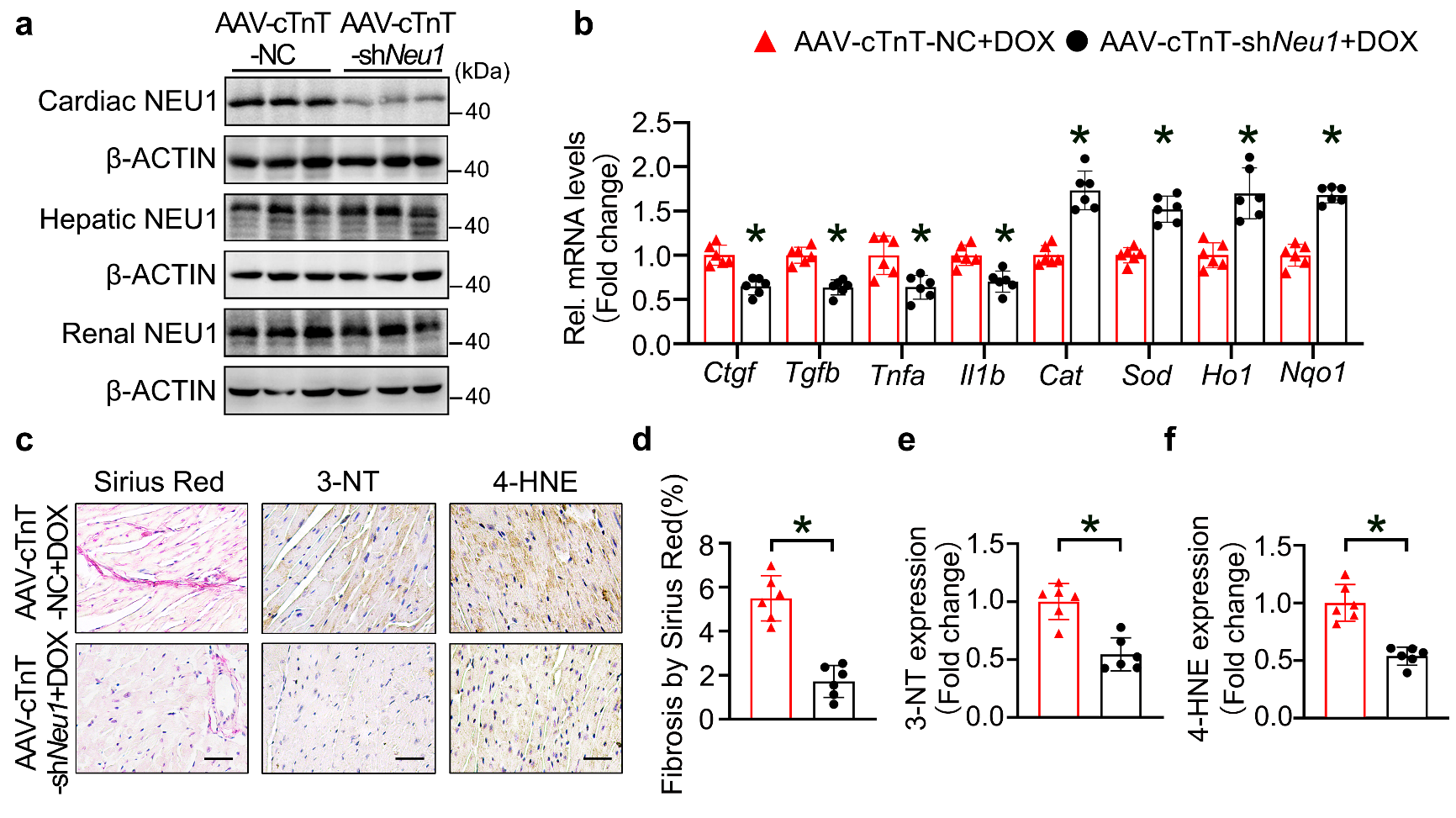
Figure S4. Cardiac-specific *Neu1* Knockdown Significantly Improved DOX-induced Cardiac Damage.** a) Representative western blot images of NEU1 in various organs of mice given AAV-cTnT-NC or AAV-cTnT-sh*Neu1*. b) The mRNA levels of *Ctgf*, *Tgfb*, *Tnfa,* *Il1b, Cat*, *Sod*, *Ho1* and *Nqo1* were determined by RT-qPCR (n=6). c-f) The representative image and quantitation of Sirius red, 3-NT and 4-HNE staining in cardiac tissue (n=6). β-ACTIN as an internal control. Data are expressed as mean±SD, Scale bar=20μm in (c), **p* < 0.05.


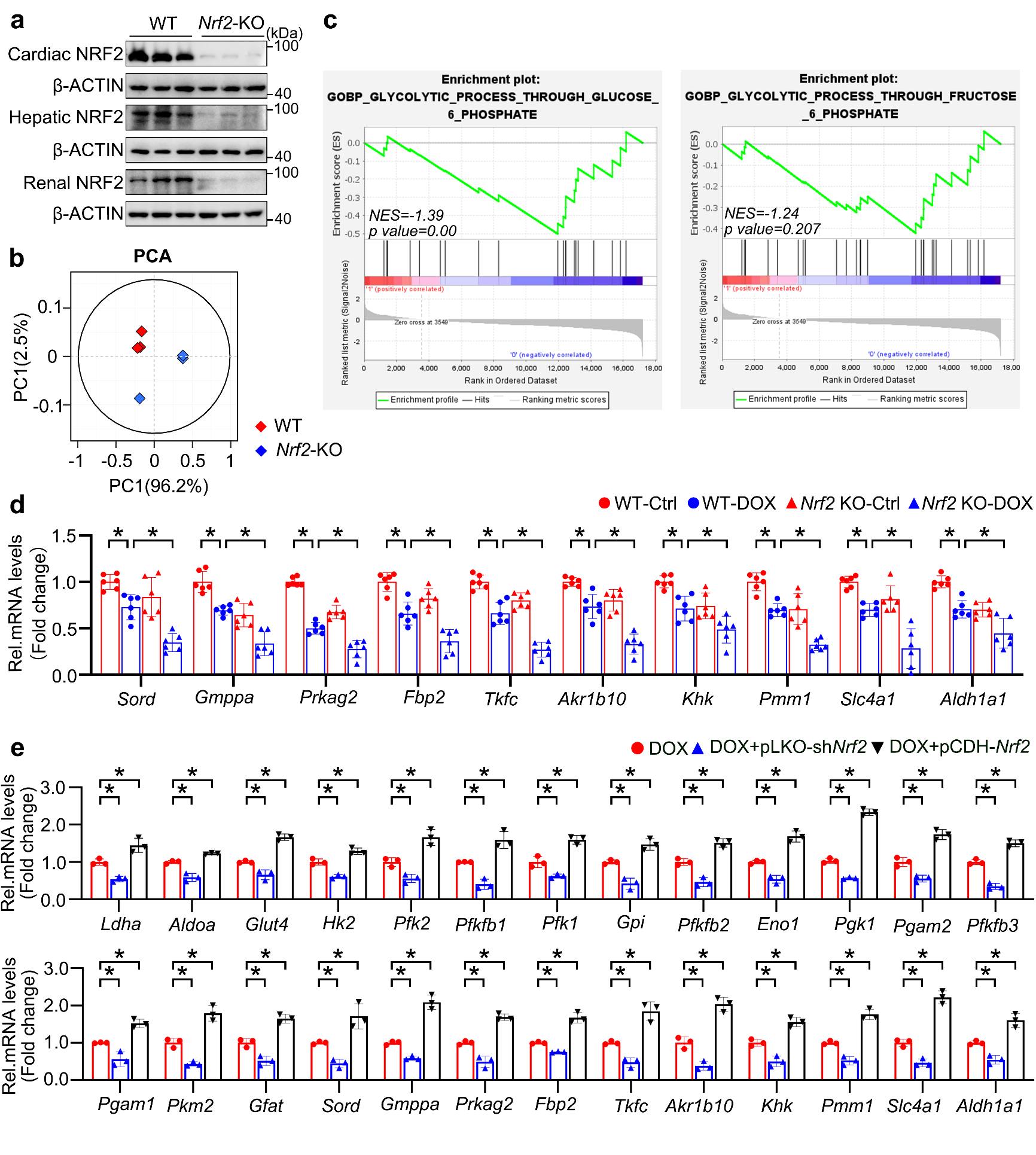


**Figure S5. NRF2 Reversed the DOX-induced Downregulation of Key Glycolytic Enzymes.** a) Representative western blot images of NRF2 in various organs of WT and *Nrf2*-KO mice. b) The principal component analysis was conducted on transcriptomic analysis of the heart from DOX-treated WT and *Nrf2*-KO mice (n=3). c) GSEA analysis of the “glycolytic process through glucose 6 phosphate” and “glycolytic process through fructose 6 phosphate”. d) Relative mRNA levels of genes related to glucose metabolism in WT and *Nrf2*-KO mice after DOX treatment (n=6). e) The mRNA levels of glucose metabolic enzymes in NRCMs transfected with pLKO-sh*Nrf2* or pCDH-*Nrf2* under DOX treatment (n=3). β-ACTIN as an internal control. Data are expressed as mean±SD, **p* < 0.05.

**
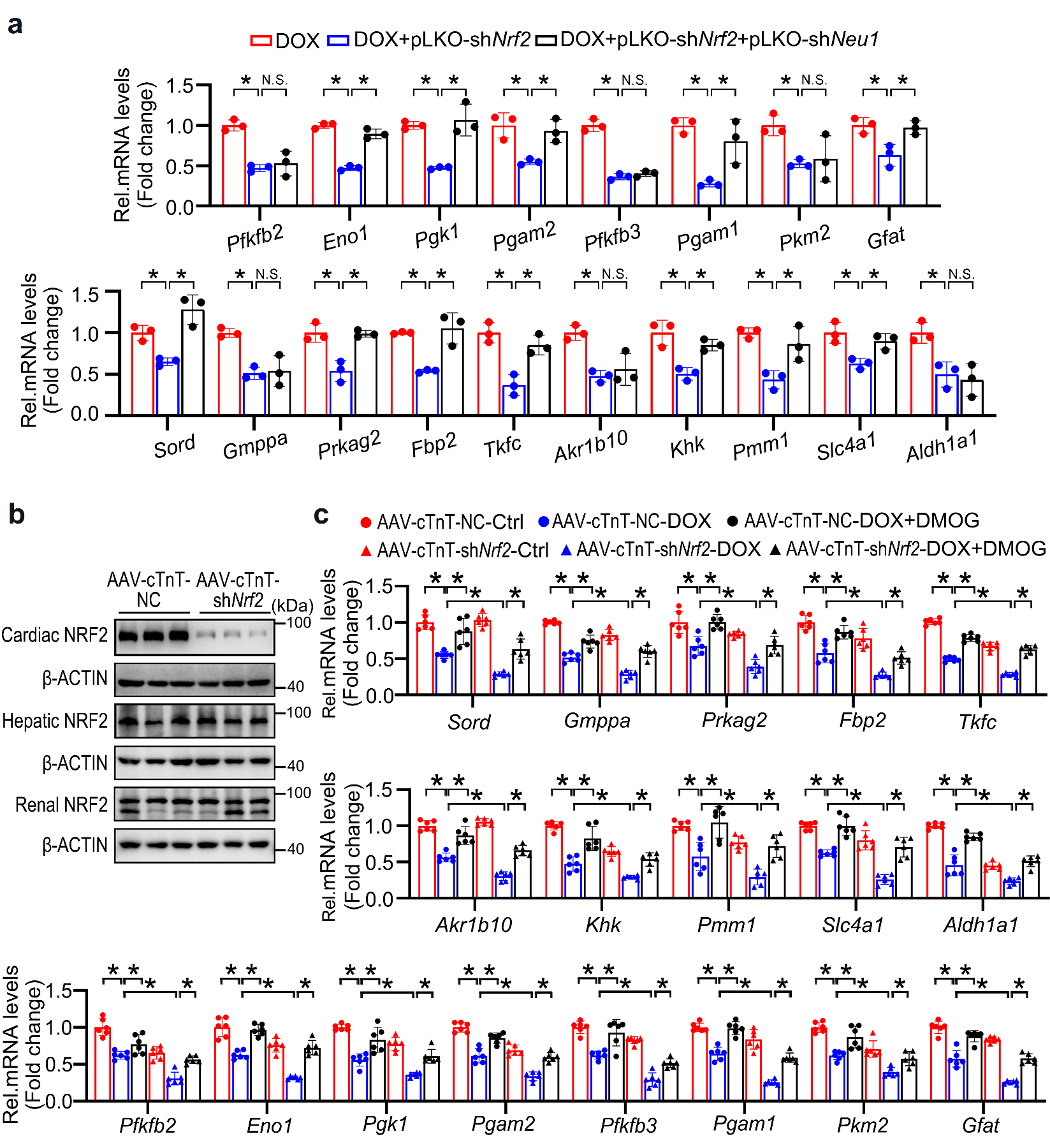
**

**Figure S6. NRF2 Modulates Glucose Metabolism through the HIF1α/NEU1 Axis.** a) NRCMs transfected with pLKO-sh*Nrf2* with or without pLKO-sh*Neu1* were treated with DOX for 24h. The relative mRNA levels were measured by RT-qPCR (n=3). b) Western blot analysis was performed to assess NRF2 protein levels in various organs of mice given AAV-cTnT-NC or AAV-cTnT-sh*Nrf2*. c) Relative mRNA levels in cardiac tissues were analyzed by RT-qPCR (n=6). β-ACTIN as an internal control. Data are expressed as mean±SD, **p* < 0.05, N.S. indicates no significance.


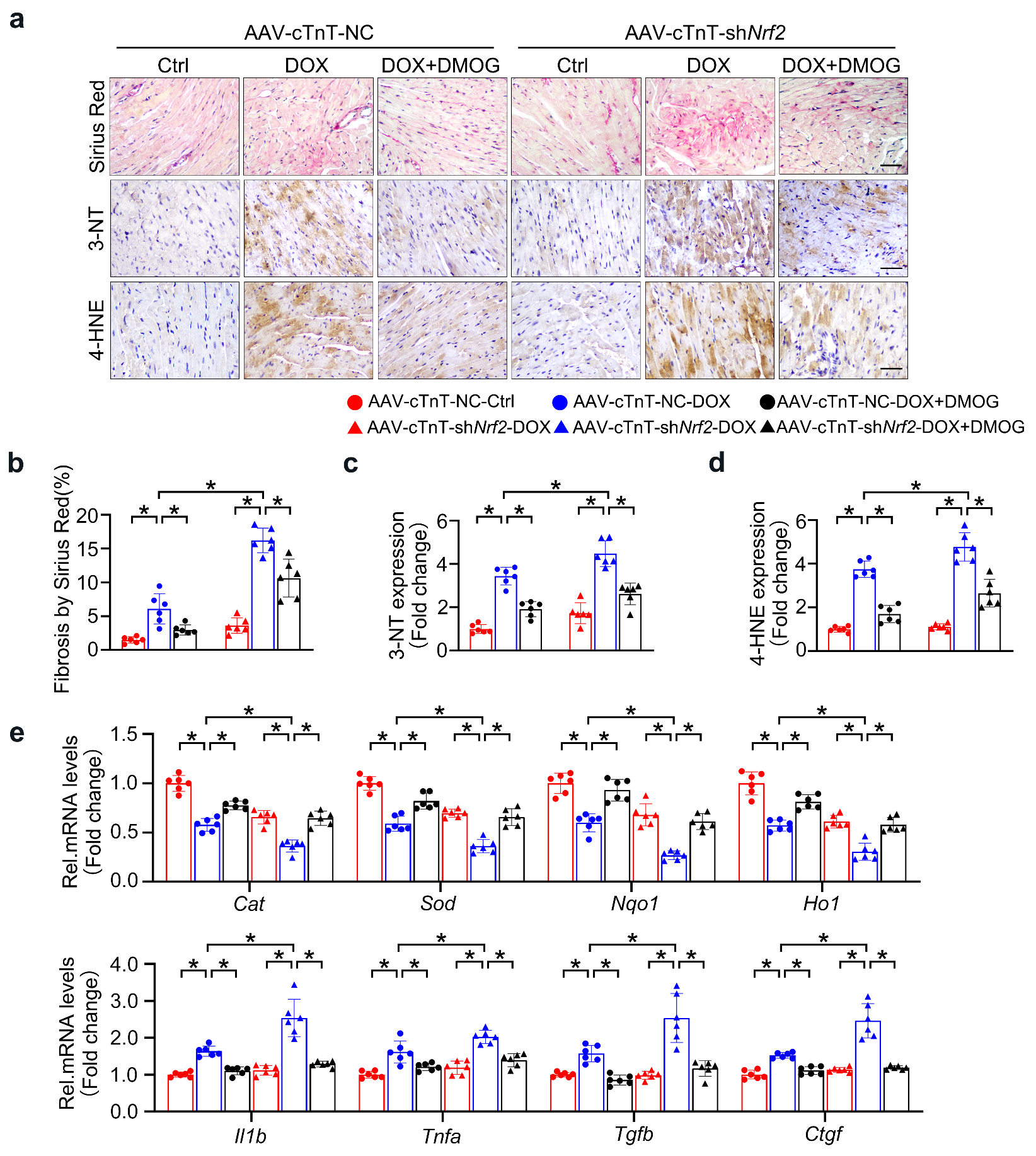


**Figure S7. The Stabilization of HIF1α Ameliorates the Myocardial Remodeling Induced by *Nrf2* Knockdown in DIC.** a-d) Representative images and quantitative analysis of Sirius red staining and IHC staining for 3-NT and 4-HNE (n=6). e) The mRNA levels of *Cat*, *Sod*, *Nqo1*, *Ho1*, *Il1b*, *Tnfa*, *Ctgf* and *Tgfb* were detected by RT-qPCR (n=6). Data are expressed as mean±SD. Scale bar =20μm in (a), **p* < 0.05.


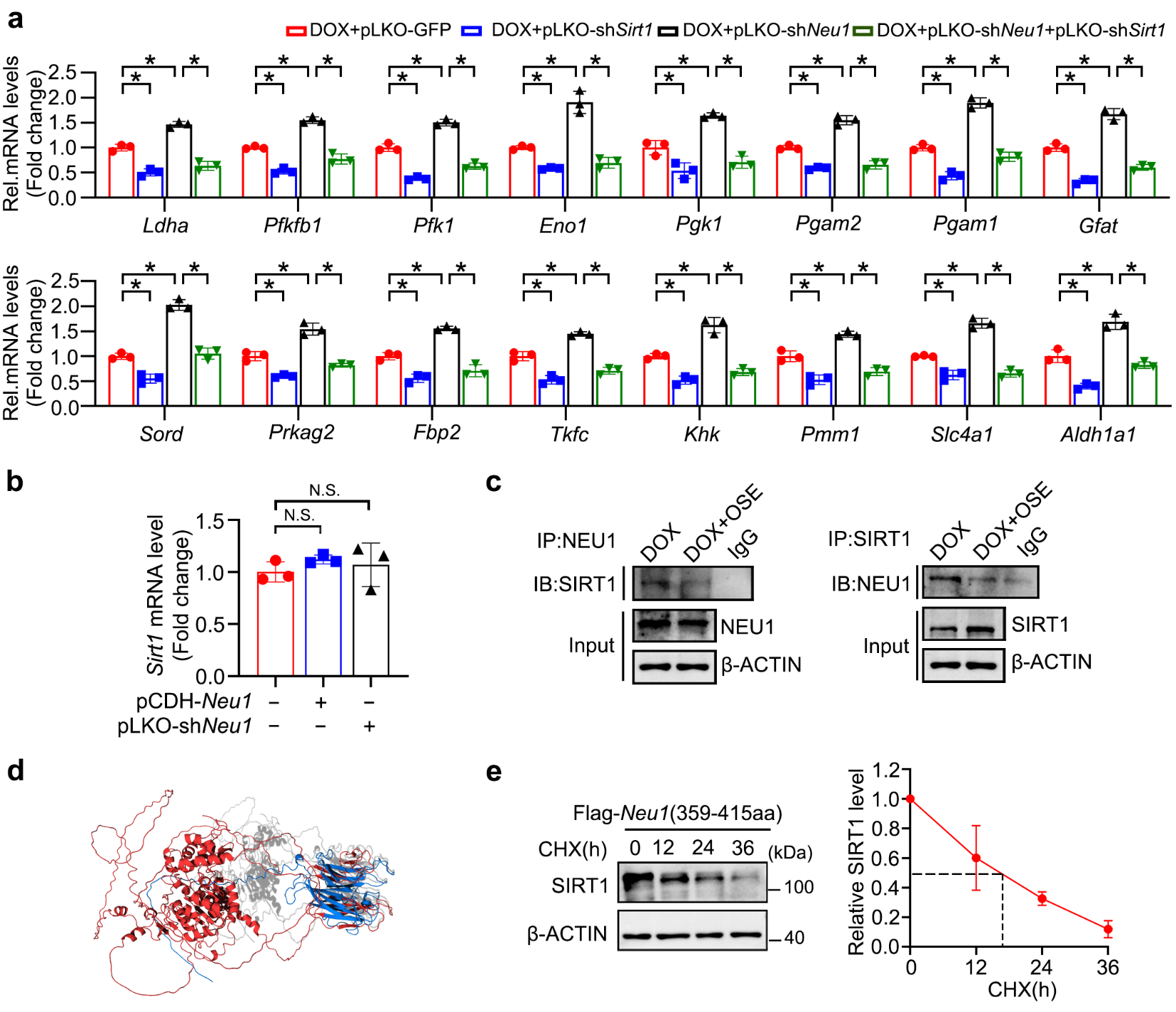


**Figure S8. NEU1 Blunts the mRNA Level of Key Glycolytic Enzymes by Suppressing SIRT1 Protein Expression.** a) Relative mRNA levels of glucose metabolic genes in NRCMs transfected with pLKO-GFP, pLKO-sh*Neu1* and/or pLKO-sh*Sirt1* under DOX treatment (n=3). b) The mRNA level of *Sirt1* in NRCMs transfected with pCDH-*Neu1* or pLKO-sh*Neu1* (n=3). c) H9c2 cells treated with 1 μM DOX with or without 10 μM OSE for 24 hours were subjected to Co-IP assay. d) Docking in Z-DOCK was employed to predict the interaction between NEU1 (blue) and SIRT1 (red). e) 293T cells were co-transfected with HA-*Sirt1* and Flag-*Neu1* containing only the C-terminal region (aa 359-415) for 48h, then the cells were treated with CHX for 0, 12, 24 or 36h. The protein level of SIRT1 was measured by western blot (n=3). β-ACTIN as an internal control. Data are expressed as mean±SD, **p* < 0.05, N.S. indicates no significance.


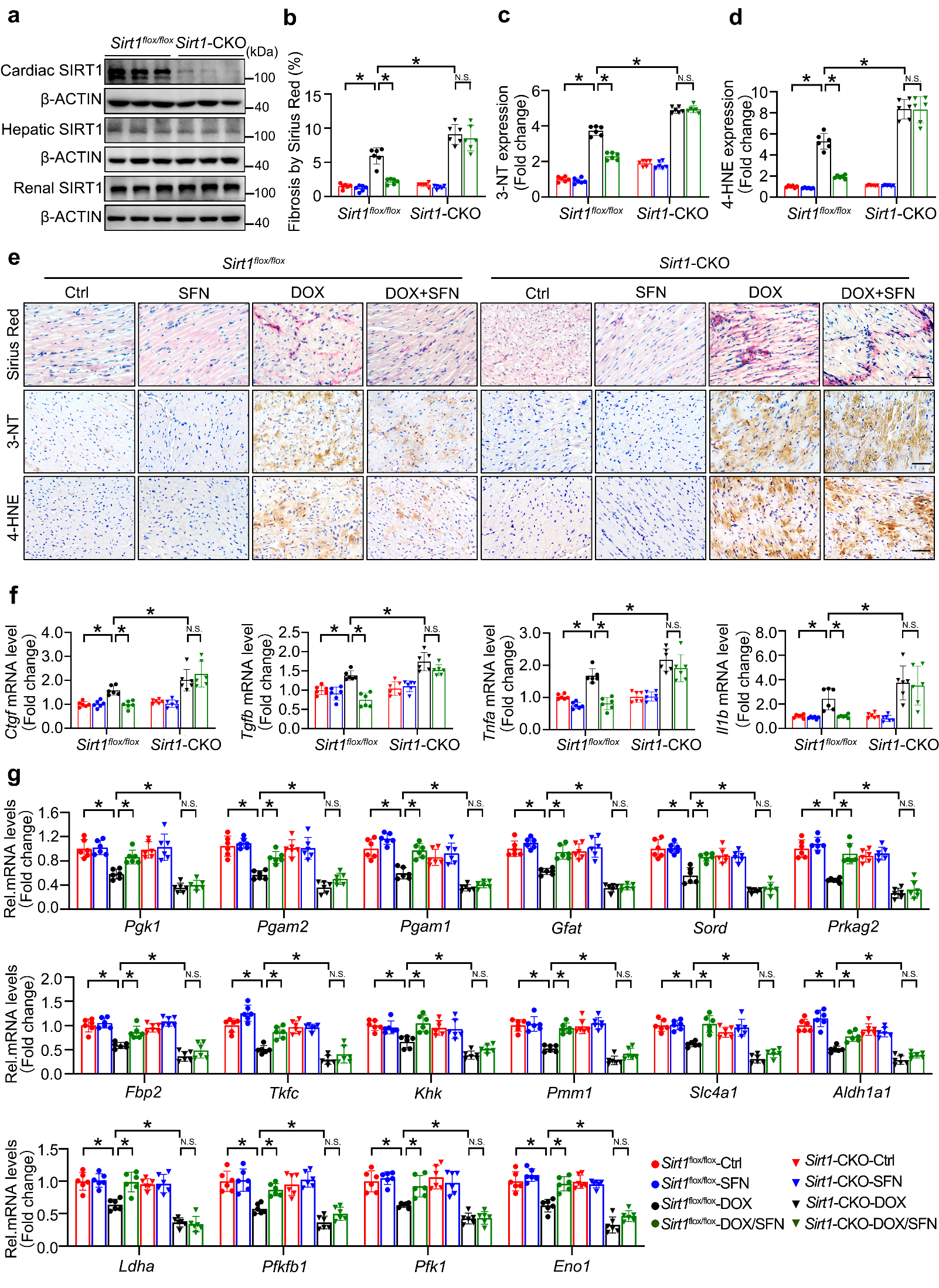


**Figure S9. Cardiac-specific Knockout of *Sirt1* Abolished the Ameliorative Effects of NRF2 Activation on Inflammation and Fibrosis in DIC.** a) Representative western blot images of SIRT1 in various organs of *Sirt1*^flox/flox^ mice and *Sirt1*-CKO mice. b-e) Fibrosis and oxidative stress were detected by Sirius Red staining and IHC staining of 3-NT and 4-HNE (n=6). f) The mRNA levels of *Il1b*, *Tnfa*, *Ctgf* and *Tgfb* were examined using RT-qPCR (n=6). g) The expression of genes related to glycolysis was determined by RT-qPCR (n=6). β-ACTIN as an internal control. Data are expressed as mean±SD. **p* < 0.05, N.S. indicates no significance. Scale bar =20μm in (e).


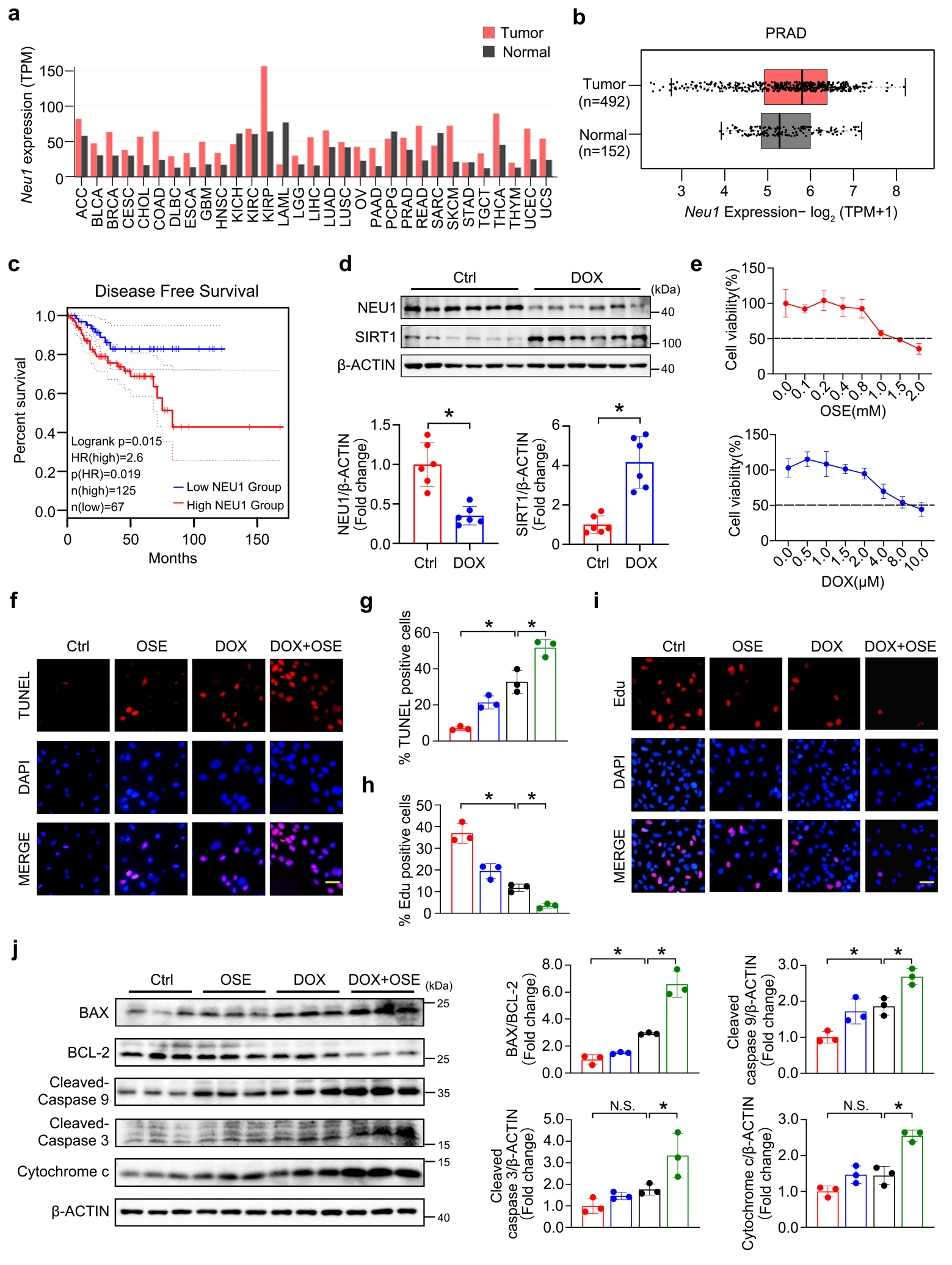


**Figure S10. Inhibition of NEU1 enhances the anticancer effect of DOX.** a) *Neu1* expression level in different cancers verified using GEPIA database. b) The expression of *Neu1* in Prostate adenocarcinoma (PRAD) (n = 492) and non-tumour tissues (n = 152) from GEPIA database. c) Disease-free survival time analysis of data from the GEPIA database. d) BALB/c-nu nude mice bearing PC-3 human prostate cancer xenografts (approximately 100 mm^3^) were treated with normal saline or DOX (5 mg/kg) via intraperitoneal injection once a week for three weeks. And the protein levels of NEU1 and SIRT1 in the lysates from xenograft tumors were examined by western blot (n=6). e) Various concentrations of DOX and OSE were applied to stimulate PC-3 cells for 24h. Then, cell viability was measured by CCK8 assay (n=3). f-i) TUNEL staining and Edu staining were conducted to evaluate the apoptosis and cells proliferation in PC-3 cells stimulated by DOX and/or OSE for 24h (n=3). j) Western blots and quantification were performed to assess BAX, BCL-2, Cleaved Caspase-3, Cleaved Caspase-9 and Cytochrome c in PC-3 cells after DOX and/or OSE treatment (n=3). β-ACTIN as an internal control. Data in (b) are presented as box-and-whisker plots displaying median and interquartile ranges, other data are expressed as mean±SD. **p* < 0.05, N.S. indicates no significance. Scale bar =10μm in (f and i).

**Reference**

[1] M. Xiao, Y. Tang, J. Wang, G. Lu, J. Niu, J. Wang, J. Li, Q. Liu, Z. Wang, Z. Huang, Y. Guo, T. Gao, X. Zhang, S. Yue, J. Gu, *Redox Biol*. **2022**, *49*, 102219.

[2] C. D. Madsen, J. T. Pedersen, F. A. Venning, L. B. Singh, E. Moeendarbary, G. Charras, T. R. Cox, E. Sahai, J. T. Erler, *EMBO Rep*. **2015**, *16* (10), 1394.

[3] M. Milkiewicz, C. W. Pugh, S. Egginton, *J Physiol*. **2004**, *560* (Pt 1), 21.

[4] M. E. Prados, A. García-Martín, J. D. Unciti-Broceta, B. Palomares, J. A. Collado, A. Minassi, M. A. Calzado, G. Appendino, E. Muñoz, *Acta Pharmacol Sin*. **2021**, *42* (7), 1124.

[5] C. Wang, W. Zhang, W. Xu, Z. Liu, K. Huang, *Clin Transl Med*. **2022**, *12* (5), e854.

[6] G. Lu, Y. Tang, O. Chen, Y. Guo, M. Xiao, J. Wang, Q. Liu, J. Li, T. Gao, X. Zhang, J. Zhang, Q. Cheng, R. Kuang, J. Gu, *J Adv Res*. **2024**.

[7] J. Wang A, Y. Tang, J. Zhang, J. Wang B, M. Xiao, G. Lu, J. Li, Q. Liu, Y. Guo, J. Gu, *Redox Biol*. **2022**, *52*, 102310.

[8] K. Dirscherl, M. Schläpfer, B. Roth Z'graggen, R. H. Wenger, C. Booy, R. Flury-Frei, R. Fatzer, C. Aloman, B. Bartosch, R. Parent, V. Kurtcuoglu, D. de Zélicourt, D. R. Spahn, B. Beck Schimmer, E. Schadde, *Scientific Reports*. **2020**, *10* (1), 4392.

[9] T. Gao, J. Wang, M. Xiao, J. Wang, S. Wang, Y. Tang, J. Zhang, G. Lu, H. Guo, Y. Guo, Q. Liu, J. Li, J. Gu, *Antioxid Redox Signal*. **2024**, *40* (10-12), 598.

[10] J. Li, Y. Tang, G. Lu, Q. Liu, Y. Guo, J. Wang, M. Xiao, T. Gao, X. Zhang, J. Gu, *J Adv Res*. **2024**.

[11] G. Lu, Q. Liu, T. Gao, J. Li, J. Zhang, O. Chen, C. Cao, M. Mao, M. Xiao, X. Zhang, J. Wang, Y. Guo, Y. Tang, J. Gu, *Nutrients*. **2022**, *14* (19).
